# From Melanoma Development to RNA-Modified Dendritic Cell Vaccines: Highlighting the Lessons From the Past

**DOI:** 10.3389/fimmu.2021.623639

**Published:** 2021-02-22

**Authors:** Mahdi Abdoli Shadbad, Khalil Hajiasgharzadeh, Afshin Derakhshani, Nicola Silvestris, Amir Baghbanzadeh, Vito Racanelli, Behzad Baradaran

**Affiliations:** ^1^Immunology Research Center, Tabriz University of Medical Sciences, Tabriz, Iran; ^2^Student Research Committee, Tabriz University of Medical Sciences, Tabriz, Iran; ^3^Istituto di Ricovero e Cura a Carattere Scientifico (IRCCS) Istituto Tumori “Giovanni Paolo II” of Bari, Bari, Italy; ^4^Department of Biomedical Sciences and Human Oncology, Aldo Moro University of Bari, Bari, Italy; ^5^Department of Immunology, Faculty of Medicine, Tabriz University of Medical Sciences, Tabriz, Iran

**Keywords:** dendritic cells, immunotherapy, melanoma development, immune checkpoints, IDO, RNA-modified dendritic cell vaccines

## Abstract

Although melanoma remains the deadliest skin cancer, the current treatment has not resulted in the desired outcomes. Unlike chemotherapy, immunotherapy has provided more tolerable approaches and revolutionized cancer therapy. Although dendritic cell-based vaccines have minor side effects, the undesirable response rates of traditional approaches have posed questions about their clinical translation. The immunosuppressive tumor microenvironment can be the underlying reason for their low response rates. Immune checkpoints and indoleamine 2,3-dioxygenase have been implicated in the induction of immunosuppressive tumor microenvironment. Growing evidence indicates that the mitogen-activated protein kinase (MAPK) and phosphatidylinositol 3-kinase/Protein kinase B (PKB) (PI3K/AKT) pathways, as the main oncogenic pathways of melanoma, can upregulate the tumoral immune checkpoints, like programmed death-ligand 1. This study briefly represents the main oncogenic pathways of melanoma and highlights the cross-talk between these oncogenic pathways with indoleamine 2,3-dioxygenase, tumoral immune checkpoints, and myeloid-derived suppressor cells. Moreover, this study sheds light on a novel tumor antigen on melanoma, which has substantial roles in tumoral immune checkpoints expression, indoleamine 2,3-dioxygenase secretion, and stimulating the oncogenic pathways. Finally, this review collects the lessons from the previous unsuccessful trials and integrates their lessons with new approaches in RNA-modified dendritic cell vaccines. Unlike traditional approaches, the advances in single-cell RNA-sequencing techniques and RNA-modified dendritic cell vaccines along with combined therapy of the immune checkpoint inhibitors, indoleamine 2,3-dioxygenase inhibitor, and RNA-modified dendritic cell-based vaccine can overcome these auto-inductive loops and pave the way for developing robust dendritic cell-based vaccines with the most favorable response rate and the least side effects.

## Introduction

Melanoma is the malignant proliferation of neural-crest-derived pigment-producing cells located in the skin, inner ear, eye, and leptomeninges (1). Among the skin cancers, cutaneous melanoma is responsible for approximately 75% of skin cancer-related death (2). The annual incidence of melanoma has risen as rapidly as 4–6%, especially among the fair-skinned populations (3). The five-year-survival rate for malignant melanoma is estimated to be 5–19% (4).

Since dendritic cells (DCs) can bridge innate and adaptive immunity, they have focal roles in developing anti-tumoral immune responses (5). Because DCs can cross-present tumor-associated antigens to CD8^+^ T cells, they are considered professional antigen-presenting cells (APCs) (6, 7). Cytotoxic T-lymphocyte-associated protein 4 (CTLA-4), programmed cell death protein 1 (PD-1) on the T-cells, and the related ligands on the DCs, i.e., CD80/86 and PD-L1/PD-L2, are the pivotal inhibitory signals that attenuate anti-tumoral immune responses (8). Targeting these inhibitory signals can pave the way for developing potent vaccines for melanoma patients (9).

The transmembrane glycoprotein mucin 1 (MUC1) is a novel tumoral antigen on melanoma cells (10). MUC1 has been implicated in the induction of immunosuppressive tumor microenvironment (11). This antigen can recruit myeloid-derived suppressor cells (MDSCs) and drive the mitogen-activated protein kinase (MAPK) and phosphatidylinositol 3-kinase/Protein kinase B (PKB) (PI3K/AKT) pathways (11–13). Furthermore, MUC1 can augment immune checkpoint axes, which can induce tolerance against tumoral cells. Moreover, MUC1 has substantially induced metastasis and tumor growth in B16 cells (10).

Recent studies have demonstrated multiple interplays between melanoma oncogenic pathways, MUC1, the abovementioned tumoral immune checkpoints, MDSCs, and indoleamine 2,3-dioxygenase (IDO). These auto-inductive loops can inhibit the development of anti-tumoral immune responses in the melanoma microenvironment. Therefore, targeting these loops can bring ample opportunity to improve the response rates of DC-based vaccines in affected patients. Furthermore, recent advances in single-cell RNA-sequencing techniques and engineered DC-based vaccines have furthered our knowledge of tumor biology and provided ample opportunity to develop potent DC-based vaccines. Identifying new biomarkers along with the previously established tumor-related antigens and genetic modification of DC vaccines might be a promising approach for the treatment of melanoma patients. This study aims to highlight the cross-talk between the main oncogenic pathways of melanoma and immunosuppressive inducer factors, i.e., tumoral immune checkpoints, MDSCs, and IDO. This study also intends to collect lessons from the RNA-modified DC vaccine studies and previous preclinical studies to improve the response rate of DC-based vaccines in melanoma patients.

## How do Melanocytes Transform Into Melanoma?

A better understanding of melanoma transformation from extracellular and intracellular view is essential for developing a potent DC-based vaccine for melanoma patients. In the following sections, we discuss the main oncogenic signaling pathways of melanoma and their associations with the tumor microenvironment.

### Melanoma Development From Intracellular View

#### Melanoma and the MAPK Signaling Pathway

Extracellular signals can initiate the MAPK pathway via binding to receptor tyrosine kinases (RTKs). The stimulation of RTKs leads to rat sarcoma (RAS) activation, the membrane-bound GTPase (14). Following the stimulation of RTKs and relocation of GDP with GTP, activated RAS propels the RAS/rapidly accelerated fibrosarcoma (RAF)/mitogen-activated protein kinase kinase (MEK)/extracellular signal-regulated kinase (ERK) cascade. Subsequently, the activated ERK stimulates the intracellular pro-growth signals (15). The BRaf, a member of the RAF family, is prone to mutation. Indeed, the BRaf accounts for 50% of melanoma. The V600E domain of mutant BRaf, which is mutant in 95% of mutant BRaf, possesses 10-fold more phosphorylation activity than the wild one (16). This high phosphorylation capability of mutant BRaf, when couples with the mutant P16/INK4a as the result of cyclin-dependent kinase inhibitor 2A (CDKN2A) mutation or phosphatase and tensin homolog (PTEN) mutation, can lead to melanoma development (17, 18).

The NRAS, the isoform of the RAS superfamily, is another oncogenic mutant in 30% of melanoma (19). Consistent with this, the RAS inhibition can repress melanoma development in zebrafish (20). The ERK signaling pathway can modulate the c-Fos, c-Myc, c-Jun, ErbB, transforming growth factor-β (TGF-β), and forkhead box O (FoxO). The cMyc regulates approximately 10~20% of cellular genes (21). The c-Myc via epithelial-mesenchymal transition induction promotes vascular mimicry, which is a crucial process for tumor development (22). In line with this, the c-Myc is associated with a poor prognosis in cancer patients (23). Furthermore, the overexpression of c-Myc represses the senescence via suppressing the P53, P-16, and retinoblastoma protein (Rb) in melanoma cells (24). The mutation of P53 and CDKN2A are commonly observed in melanoma cells (25). Upon the oncogenic stress, P53, as the guardian of the genome, inhibits the cyclin-dependent kinase/cyclin D complex via P21 expression. This inhibition halts cellular proliferation (26). The Rb is another tumor suppressor, which can bind to E2F and abort cellular replication. However, when the cyclin-dependent kinases 4, 6/cyclin D complexes phosphorylate the Rb, Rb releases the E2F, which drives the cellular replication (27). The P-16, via repression of cyclin-dependent kinases/cyclin D complexes, mediates the cross-talk between the P53 and Rb (28).

**Figure 1:**
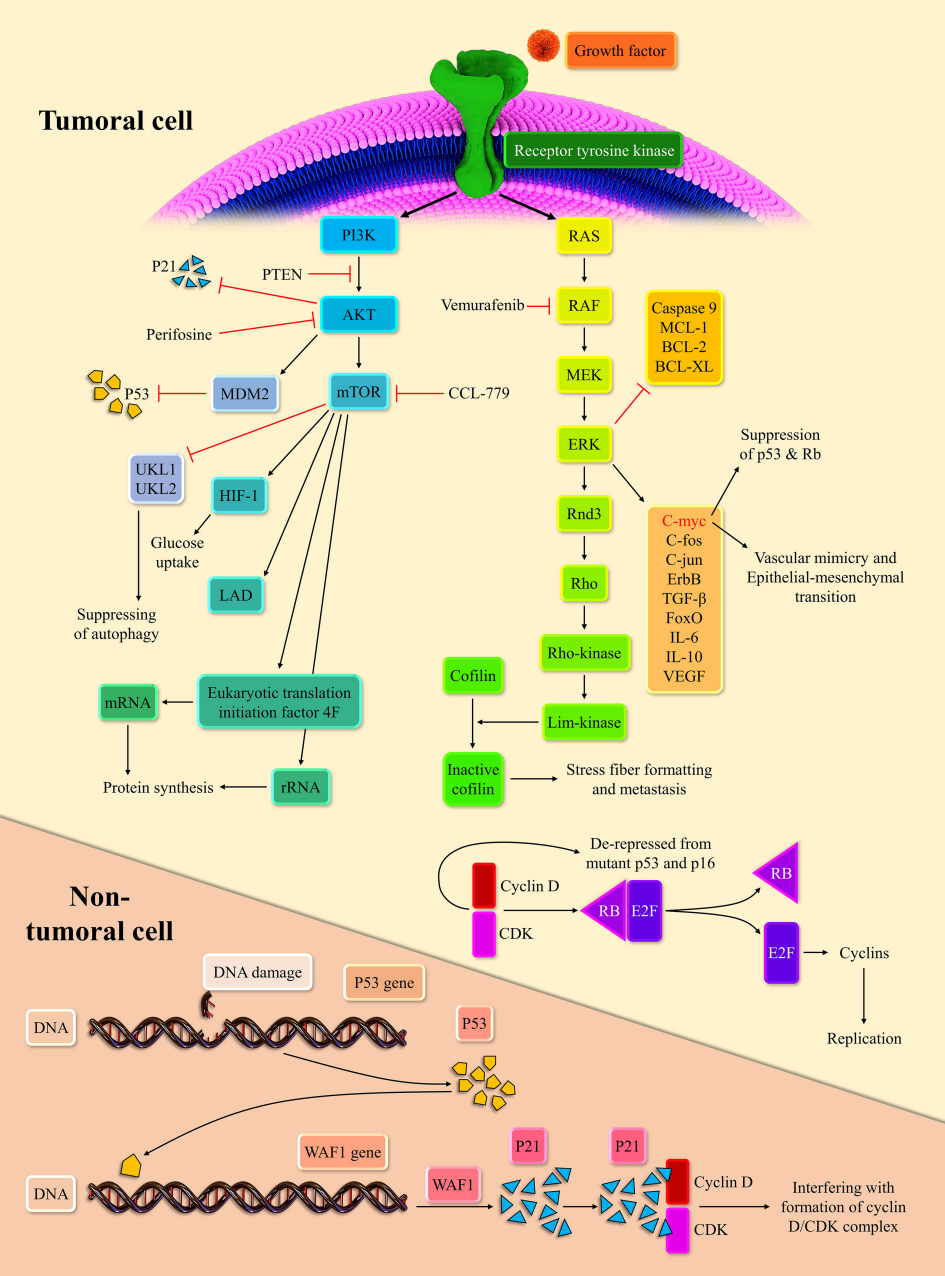
Two main intracellular carcinogenic pathways of melanoma and their inhibitors.

The ERK stabilizes dual-specificity phosphatases 5, which represses the ERK signaling pathway (29). Since the activation of the ERK signaling pathway is needed for the entry of cells to the S-phase, its inactivation has been associated with decreased cell replication (30). The ERK signaling pathway via the establishment of ERK/c-Fos/Fra-1/cyclin D and ERK/c-Myc/cyclin D upregulates the cyclin D level at the G1/S checkpoint (31, 32). At the growth 2/mitosis (G2/M) checkpoint, the ERK signaling pathway can increase the cyclin B expression (33). The up-regulation of cyclin D and cyclin B drives the cellular replication robustly. The ERK signaling pathway also downregulates the expression of anti-apoptotic agents from the apoptosis perspective, e.g., myeloid cell leukemia-1 (MCL-1), B-cell lymphoma 2 (BCL-2), and B-cell lymphoma-extra-large (BCL-XL), and inhibits the caspase 9 (34, 35). However, the ERK signaling pathway increases the P53 level via inhibition of murine double minute 2 (MDM2) with P14 (36). As mentioned earlier, the P53 mutation is predominant in various cancers; thus, this mechanism fails to induce senescence and cellular death. This intertwined ne, leading to cellular senescence, is demonstrated in Figure 1.

#### Melanoma and the PI3K/AKT Signaling Pathway

The PI3K/AKT/mechanistic target of rapamycin (mTOR) pathway is another crucial pathway in melanoma development. RTKs and activated Ras can lead to the PI3K activation. Afterward, the activated PI3K stimulates the AKT (37). Since the PTEN can regulate the AKT activation, the PTEN mutation, observed in 10–30% of melanoma cell lines, can lead to stimulation of the PI3K/AKT/mTOR pathway (17). Based on a report, the AKT/mTOR activation is noticeable in approximately 70% of melanoma patients (38). Dysregulated AKT can also activate the MDM2, which ultimately leads to the degradation of P53 (39). Indeed, dysregulated AKT suppresses the P53 expression and denatures the P21 (40). The P21 denaturation, via the cyclin-dependent kinase (CDK)/cyclin de-repression, can increase cell replication rate. This pathway can also upregulate the hypoxia-inducible factor 1 alpha and enhance the glucose uptake (41). Due to fluctuating tumoral microenvironment and hypoxia, metabolic reprogramming to anaerobic glycolysis is essential to tumoral survival. The mTORC1 activation can increase the lactate dehydrogenase expression in hypoxia-inducible factor 1 alpha-dependent-manner (42).

Since the mTOR can repress the unc-51 like autophagy activating kinase 1 (UKL1) and UKL2, it can inhibit autophagy (43). Autophagy is the last resort for the cells that blocks the transformation of cells into tumor cells (44). Due to the anti-apoptotic effect of the mTORC1, it can facilitate tumoral development. In terms of protein synthesis reprogramming, the mTOR is well-established in ribosomal RNA (rRNA) upregulation (45). The mTOR promotes eukaryotic translation initiation factor 4F complex's conformation, facilitating the messenger RNA (mRNA) translation (46).

Overall, these preliminary findings have raised the notion that suppressing these pathways can eliminate melanoma development; however, the subsequent adverse side effects have posed daunting challenges for this approach (see below).

### Melanoma Development and the Tumor Microenvironment

#### PD-L1 and Its Association With the MAPK and PI3K Signaling Pathways in Melanoma

The tumor microenvironment has substantial roles in determining the fate of cancer cells. Indeed, the balance between the stimulatory and inhibitory signals can determine the direction of anti-tumoral immune responses against tumor antigens. Immune checkpoints are the well-established inhibitory axes that can repress the T-cell mediated anti-tumoral immune responses and promote tumor growth (47).

The PD-L1/PD-1 axis is one of the well-known immune checkpoint axes. The PD-1, as a transmembrane protein, can be expressed on the T-cells and natural killer cells (48). Moreover, the PD-L1 can be expressed both on the infiltrating DCs and on the tumoral cells, e.g., melanoma cells. Of interest, the cardinal portion of melanoma tissues is highly PD-L1 expressed tissues (49). In line with this, Kleffel et al. have also demonstrated that melanoma cells can express tumoral PD-1 on their own cell surface to shield tumor cells from anti-tumoral immune responses (50). Mayoux et al. have demonstrated that PD-L1 can be more expressed on the peripheral and tumoral-associated DCs than CD80. Indeed PD-L1 antibody administration, via interfering with the cis interaction between PD-L1 and CD80, reestablishes anti-tumoral immune responses (51).

Although tumoral PD-L1 expression has been shown to promote cancer development, higher PD-L1 expression has not been correlated with inferior overall survival and progression-free survival in melanoma patients (52, 53). This controversy might stem from the considerable heterogenicity between those included studies; the previous history of patients' treatment and not unified cut-off value are only two examples of the existent heterogenicity among the included studies of that meta-analysis (53). In line with preclinical findings, another meta-analysis has indicated that the tumoral PD-L1 expression can promote metastasis in melanoma patients (54, 55).

The cross-talk between tumoral PD-L1 and the MAPK and PI3K/AKT might be one reason for impeding anti-tumoral immune responses despite the continuous tumor growth and progression. Atefi et al. have reported that the activation of the PI3K/AKT signaling pathway can increase the expression of tumoral PD-L1, resulting in tumor development (56). Furthermore, the stimulation of the MAPK signaling pathway can lead to tumoral PD-L1 expression in melanoma patients with BRaf-resistant therapy (57). In melanoma patients with positive baseline PD-L1, unlike melanoma patients with negative baseline PD-L1, BRaf inhibitors substantially downregulate the PD-L1 expression (58). Consistent with that, Sumimoto et al. have elucidated that BRaf mutation causes the immune evasion of melanoma cells. Besides, the BRaf inhibition downregulates interleukin (IL)-10, IL-6, and vascular endothelial growth factor (VEGF) (59). In melanoma cells without baseline expression of PD-L1, the tumoral PD-L1 expression does not correlate with the MAPK pathway's status (60). However, it has been reported that the inhibition of the MAPK pathway can upregulate the PD-L1 expression, decrease IL-6 and IL-8 secretion, and increase the tumor-infiltrating lymphocytes in melanoma patients (61).

**Figure 2:**
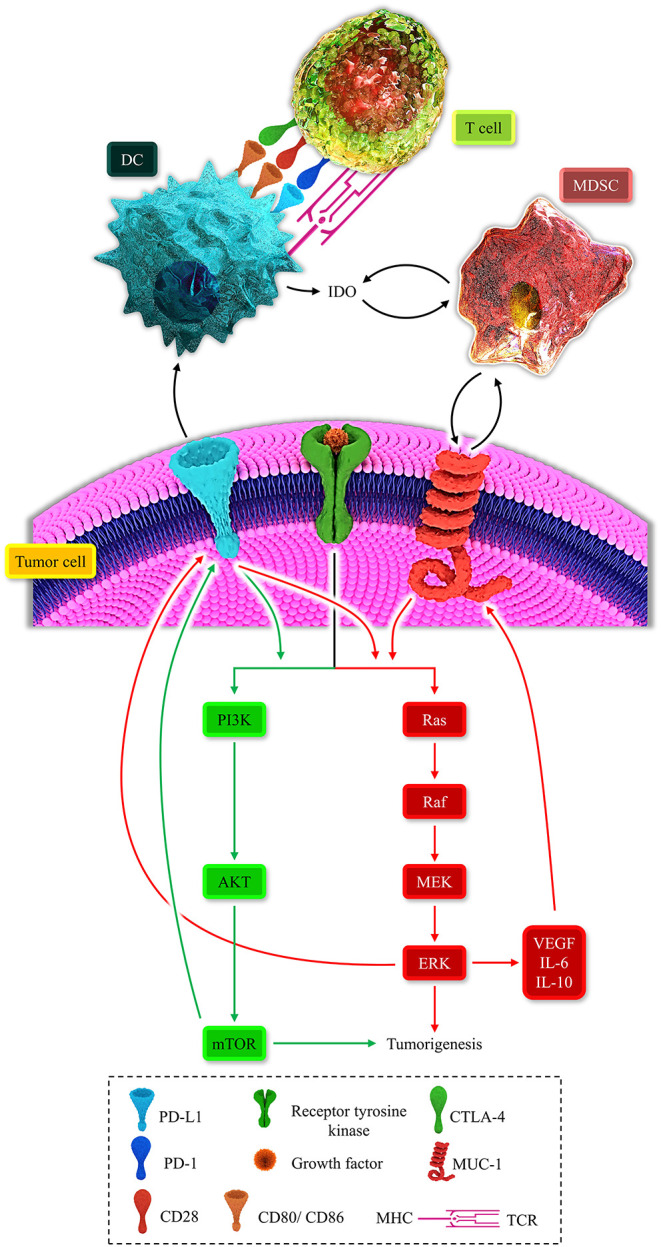
Represents the multiple positive-loops between programmed death-ligand 1 (PD-L1), cytotoxic T-lymphocyte-associated protein 4 (CTLA-4), mucin 1 (MUC1), myeloid-derived suppressor cell (MDSC), dendritic cell (DC), T-cell, the MAPK signaling pathway, and the PI3K/AKT pathway. The immunosuppressive microenvironment is partially due to these unfavorable auto-inductive loops. The PD-L1/PD-1 axis is a well-established culprit of impeding anti-tumoral immune response development. The CTLA-4/CD80, CD86, another immune checkpoint axis, is responsible for suppressing the anti-tumoral immune response. The PD-L1 on the tumoral cell surface can impede the development of the anti-tumoral immune response and stimulated the PI3K/Akt and MAPK signaling pathways. MUC1 has the focal role in the recruitment of MDSC and the propelling of the MAPK pathway. The MAPK signaling pathway is also responsible for the overexpression of MUC1. Thus, these positive loops can substantially induce an immunosuppressive micro-environment. Therefore, these two major recognized signaling pathways can induce tumorigenesis and shield the tumoral cells from anti-tumoral immune responses.

Besides melanoma, abundant studies reveal the correlation between the MAPK-PI3K/AKT oncogenic pathways and PD-L1 expression. In lung adenocarcinoma cells, the activation of the MAPK pathway upregulates the tumoral PD-L1 expression. Moreover, the inhibition of the MAPK pathway downregulates PD-L1 without affecting the major histocompatibility complex (MHC) class I level (62). In non-small-cell lung carcinoma, the stimulation of the MAPK signaling pathway can increase tumoral PD-L1 expression (63). In triple-negative breast cancer, the activation of the MAPK pathway has been associated with the upregulated tumoral PD-L1 expression (64). In glioma, the inhibition of the PTEN post-transcriptionally upregulates the tumoral PD-L1 expression (65). In estrogen receptor α-positive endometrial and breast cancer cells, 17β-estradiol, via the PI3K/AKT signaling pathway, increases the PD-L1 expression (66). In melanoma, the PTEN loss has been associated with upregulated expression of tumoral PD-L1 and secretion of VEGF, IL-6, and IL-10 (67). Furthermore, the interaction between PD-1/PD-L1 can stimulate the MAPK and PI3K/AKT signaling pathways, which results in an establishment of auto-inductive loops (68). Overall, there is a positive loop between the MAPK and PI3K/AKT pathways and tumoral PD-L1 expression, leading to tumor growth (56, 57, 68) (Figure 2).

#### CTLA-4 and Its Association With the MAPK and PI3K Signaling Pathways in Melanoma

TLA-4 was initially shown on the surface of T-cells. However, recent findings have identified them on the tumoral cells, e.g., melanoma and luminal B breast cancer cells (69, 70). Several mechanisms have been linked to the CTLA-4 inhibitory function. The CTLA-4 via competition with CD28 to binding with CD80/CD86 inhibits the secondary signal. The inhibition of the second signal represses anti-tumoral immune responses of T-cells (71). CTLA-4 can also inhibit the IL-2 gene expression and prevent the cell cycle via downregulation of cyclin D3 and cdk4/6 (72). Since CTLA-4 can regulate the activity of T-cells and APCs, e.g., DCs, this immune checkpoint can induce immune tolerance, facilitating tumor evasion (73). The regulatory effect of CTLA-4 is subjected to a negative feedback loop. The stimulatory signals via TCR complexes and CD80/CD86: CD28 upregulates the CTLA-4 density on the T-cells receptor (74). Consistent with this, CTLA-4 overexpression on tumor-infiltrating lymphocytes has been considered as a red flag for melanoma patients [hazard ratio (HR): 7.962, 95% CI: 1.832–34.598, *P*-value = 0.006] (75).

Besides the expression of CTLA-4 on immune cells, the CTLA-4 can also be highly expressed on the cell surface of human melanoma cells (70). The increased density of CTLA-4 on the melanoma cells is due to the activation of the JAK1/2 pathway, mediated by interferon-gamma (IFN-γ). Besides the positive association between the MAPK signaling pathway with tumoral PD-L1, the MAPK signaling pathway can also stimulate the expression of CTLA-4 on the melanocytes (70). In melanoma cells, the tumoral expression of CTLA-4 can maintain the stem cell feature of melanoma and confer anti-apoptotic features to the tumor (76). With the recent findings of tumor CTLA-4 and PD-1 expression on melanoma, the cross-talk between the CTLA-4/PD-1 and MAPK-PI3K/AKT signaling pathways deserves further investigation.

#### MUC1: A Novel Antigen in Melanoma?

Since tumor-related antigens are not present overtly on the non-tumoral cells, immunotherapy can efficiently target these aberrant antigens (77). Among the well-established tumor-related antigens, MUC1 might be a promising candidate in vaccine development (78–82). Scheikl-Gatard et al. have confirmed nine human leukocyte antigen (HLA)-restricted peptides of MUC1, which is indicative of its acceptable level of immunogenicity (83). Consistent with this, MUC1 has shown promising results in cancer vaccines. In non-small cell lung cancer patients resistant to standard anticancer therapies, a DC-based vaccine for MUC1 has improved the patients' survival (84). In patients with non-small cell lung cancer, MUC1 mRNA vaccines have been well-tolerable and induced specific-immune responses in 84% of affected patients (81).

Although normal epidermal cells do not express MUC1, normal MUC1 can defend against infective pathogens in the digestive tract (85, 86). The aberrant MUC1 is present in melanoma and has been implicated in its metastasis (10). Compared to normal human epidermal melanocytes, MUC1 has been substantially expressed in the A375, B16, CHL-1, SK-MEL-2, SK-MEL-5, B16BL6, and B16 cells. Silencing MUC1 expression has decreased tumor migration via activation of the PI3K/AKT signaling. Moreover, targeting MUC1 has substantially reduced the metastasis of melanoma (10). In contrast, the MUC1 can inhibit the cyclin D and c-Myc levels, resulting in the cellular cycle arrest in MUC1-transfected tumoral cells. Similar results have been reported following the inoculation of tumoral cells in the animal model. However, that study did not determine the precise interaction between MUC1 and specific immune cells, i.e., MDSCs, and DCs. Moreover, MUC1 transfection to the tumoral cells might not serve as tumoral MUC1 (87). Since aberrant MUC1 can facilitate the expression of ATP-binding cassette transporter B1, it can develop chemoresistant and drives the carcinogenic pathway. Furthermore, it is crucial to develop an immunosuppressive microenvironment (11, 88, 89). In terms of intracellular interactions, the cytoplasmic tail of MUC1 increases the activation of the MAPK signaling pathway (90). Moreover, MUC1 establishes various auto-inductive loops with MDSCs in the tumor microenvironment, which recruit the MDSCs in the MAPK and PI3K/AKT pathways manner. MUC1, via stimulation of oncogenic pathways like the MAPK pathway, can increase the c-Myc level in nuclear factor kappa-light-chain-enhancer of activated B cells (NF-κB) p65 manner, which results in immune evasion (91). In line with this, MUC1 suppression has been associated with the downregulation of PD-L1 on non-small-cell-lung cancer and has augmented the anti-tumoral immune response (92). As discussed earlier, PD-L1 expansion induces an immunosuppressive tumoral microenvironment and drives the MAPK and PI3K/AKT pathways (59), which leads to the expression of MUC1 and the establishment of an auto-inductive loop (93, 94). These positive feedback loops facilitate tumoral development, metastasis, and impairment of anti-tumoral immune responses. Although MUC1 upregulates the PD-L1 expression (92), there is no detailed investigation about the direct effect of PD-L1 on MUC1 expression. However, based on separate studies, PD-L1 can upregulate MUC1 via the oncogenic pathways (10, 56, 61).

#### IDO and Its Roles in Inducing Immunosuppressive Microenvironment

IDO can transform the pro-inflammatory tumor microenvironment into an immunosuppressive tumor micro-environment. Tumoral cells increase IDO level, leading to the inhibition of effector T-cells (95). Furthermore, some types of DCs can promote the level of IDO in the tumoral microenvironment and tumor-draining-lymph-nodes (96). This enzyme can induce tolerance to tumoral antigens and pave the way for tumor development (97). In the tumoral context, the cardinal functions of IDO are tryptophan depletion and kynurenine upregulation, which tilt the balance to immunosuppression (98). Recently, Shang et al. have demonstrated that the tumoral IDO knockdown can substantially decrease the PD-1 expression on T-cells and increase the IL-2 plasmatic level in lung cancer-bearing mice (99). In line with that, it has been estimated that 37% of melanoma specimens express IDO, which its expression is substantially associated with tumoral PD-L1 expression and nodal metastasis (100). A recent study on melanoma has highlighted a remarkable association between the plasmatic kynurenine/tryptophan ratio with poor overall survival and resistance to nivolumab, PD-1 targeting antibody (101). In melanoma patients, the expression of IDO has been associated with inferior overall survival and progression-free survival. Moreover, the IDO expression in sentinel lymph nodes has been associated with CTLA-4 expression on regulatory T cells (T-reg) cells (102). Consistent with this, the IDO expression in sentinel lymph nodes and primary melanoma has been associated with poor overall survival. Moreover, there has been a strong association between IDO expression in sentinel lymph nodes with the IDO expression in the peritumoral stroma (103). Of interest, the high expression of IDO has been significantly associated with tumoral PD-L1 expression (104).

MDSCs can also contribute to the IDO upregulation, and in return, IDO facilitates the recruitment of MDSCs. Moreover, the PI3K/AKT signaling pathway can pave the way for recruiting MDSCs in the tumor microenvironment (105). Indeed, there are multiple auto-inductive loops between the MAPK-PI3K/AKT signaling pathways and MDSCs. These positive loops repress the anti-tumoral immune response (11). Furthermore, MDSC expansion can fail immune checkpoint blockade approaches (106). Therefore, positive immunosuppressive loops can be established between MDSCs, IDO, oncogenic pathways, and immune checkpoints (Figure 2).

## DCs in the Tumor Microenvironment and DC-Based Vaccine Development

### DCs in the Tumor Microenvironment of Melanoma

Although recent findings have indicated that there is a synapse between various DCs to share tumor antigens (107), human DCs can be divided into plasmacytoid DC (pDC), conventional DC (cDC), and monocyte-derived DCs (MoDC) subsets. The cDCs can also be subdivided into cDC1 and cDC2 subgroups (108–110).

PDCs can impede the anti-tumoral immune responses of killer (NK) cells and T-cells via PD-L1 overexpression (111). Moreover, IL-10 has been considered one of the main culprits of inducing the immunosuppressive tumor microenvironment via pDCs. The probable reason for this effect might be stemmed from the impaired function of INF-α, which IL-10 can cause in the tumor microenvironment (112). Moreover, pDC can induce the tumoral-secreted IL-10 in hepatocellular carcinoma, which might establish an immunosuppressive auto-inductive loop (113). Consistent with this, it has been shown that a higher circulating pDC and MDSCs have been associated with a poor prognosis in melanoma patients (HR = 4.42, 95%CI: 1.27–15.33, and HR = 4.98, 95%CI: 1.50–16.67, respectively).

CDC1 is assigned to deliver antigens to the lymph node, recruit the effector T-cells via expression of chemokine (C-X-C motif) ligand 9 (CXCL9) and C-X-C motif chemokine ligand 10 (CXCL10), and stimulate T-cells and NK cells via secretion of IL-12 (114, 115). In animal models, NK cells can induce chemokine (C-C motif) ligand 5 (CCL5) and lymphotactin (XCL1) and recruit cDC1 to the tumor microenvironment; however, tumoral prostaglandin E2 (PGE2) can inhibit this anti-tumoral positive loop (116). Consistent with this, targeting PEG2 has been associated with increased responsiveness to immunotherapy in BRaf and NRas melanoma cells (117). In line with that, the levels of cDC1 and NK cells have been associated with a better prognosis in melanoma patients. Moreover, these cells can increase the responsiveness of PD-1 immunotherapy (118). CDC1 has been associated with the cross-presentation of antigens to develop anti-tumoral immune responses of CD8^+^ T cells (119). However, recent data have indicated that cDC1 can stimulate both CD4^+^ and CD8^+^ T cells (120). Furthermore, TGF-β, which MUC1 can facilitate its expression, can impair this cross-presentation (11, 121). In line with this, a recent study has shown that TGF-β can promote melanoma metastasis in advanced stages (122). Indeed, the immunosuppressive microenvironment can inhibit the migration and stimulation of cDC1 and promote MDSC recruitment. Recent studies have shown that IL-10 is an essential factor in limiting the cross-presentation of cDC1 following cancer vaccination (123). Moreover, recent data have indicated that PD-L1 can be overexpressed on cDC1 and considerably attenuate the anti-tumoral immune responses (124).

Although cDC2 is mainly responsible for the activation of CD4^+^ T cells, recent data have shown that cDC2 is also involved in priming CD8^+^ T-cells (125, 126). CDC2 can secrete IL-23, IL-1β, and IL-6, resulting in the differentiation of Th-17 cells. The Th-17 differentiation has been accompanied by IL-17 secretion and RORγt upregulation. Moreover, this subtype, like cDC1, has demonstrated substantial migratory ability to lymph nodes (125). Although cDC2 can reduce the recruitment of MDSCs to the tumor microenvironment, the cDC1 vaccines have more potent than the cDC2 vaccine in B16 melanoma tumor-bearing animal models (125). However, other findings have indicated both anti-tumoral and pro-tumoral proprieties of cDC2 (127). Overall, our knowledge about this subtype is still growing, and its functional role needs further research.

MoDCs are commonly developed following inflammation and can promote Th-1 and Th-17 development (128). Although MoDCs can be straightforwardly obtained from the blood of affected patients, their limited migratory ability has not led to the desired results. Indeed, the MoDCs of cancer patients cannot substantially produce IFN-γ. The underlying reason for this might be attributable to the remarkable expression of IL-6 (129). Oosterhoff et al. have implicated the STAT3 and the p38 MAPK signaling as the main culprits of IL-10, IL-6, PGE2, TGFβ expression in MoDCs. Indeed, signal transducer and activator of transcription 3 (STAT3) inhibition has been associated with a decreased level of IL-6 in melanoma and glioma cell lines (130). MoDCs have shown promising results in infectious diseases. In infection with Toxoplasma gondii cysts, NK cells can produce IFN-γ can pave the way for the MoDC development, which is an abundant source of IL-12 secretion (131). In lymphocytic choriomeningitis virus infection, MoDCs are the cornerstone of developing CD8^+^ memory cells (132).

### Novel Direction for DC-Based Vaccine: RNA-Modified Dc Vaccines

The anti-tumoral vaccines aim to expand the specific effector lymphocytes in the tumoral microenvironment. These clonal T-cells efficiently detect the specific targeted tumor-related antigen and develop an anti-tumoral immune response. DCs, as the professional APC, are the pivotal cells in the activation of specific T-cells (6, 7). Indeed, they uptake the antigens via danger-associated molecular patterns and pathogen-associated molecular patterns, process them in small peptides, and present MHC-peptides to CD8^+^ and CD4^+^ cells. The detailed mechanism of each stage is reviewed elsewhere (133). However, “training” of DCs against a wide variety of tumoral antigens, as DC-based vaccines, does not require the identification and uptake of tumoral antigens by trained DCs (134). Indeed, one of the advantages of DC-based vaccines over other approaches is their ability to recognize a wide range of antigens, limiting the chances of tumor evasion from immunological escape (135).

The fundamental concept in developing DC-based vaccines was the training of DCs with tumoral antigen in *ex vivo*. Then, the trained DCs were administered to the affected patients with cancer (136). Genetic reprogramming has opened a new era in the field of cancer vaccines. This approach includes the genetic modulation of DCs via mRNA and small inhibitory RNA (siRNA), which can improve the ability of DCs to present tumor-related antigens and attenuate the immunosuppressive tumor microenvironment.

#### mRNA

The application of the single-cell RNA-sequencing techniques might be a promising approach to classify the tumoral antigens and the tumor microenvironment (137). The first step in applying single-cell RNA-sequencing is distinguishing the tumoral cells from non-tumoral cells, which might seem to be challenging. However, copy-number variations may help distinguish the neoplastic cells from non-neoplastic cells based on their genome lesions (138). After identifying the neoplastic cells, the search for the tumor-related antigens might be valuable for targeting tumoral cells. For instance, Tirosh et al. (137) have furthered our knowledge about the oligodendroglioma and identified a rare population of undifferentiated cells that propagate the tumor growth (137). Since the tumoral cells are continuously progressing, the single-cell RNA-sequencing can pave the way for identifying new tumor-related antigens. Ramsköld et al. (139) have identified a different set of gene expression among melanoma cell lines based on the data of the mRNA-sequencing protocol (Smart-Seq), which can be used as target biomarkers in cancer therapy. As aforementioned, DC-based vaccines can be sensitive to a mixture of antigens, thus applying the data from the single-cell RNA-sequencing technique, besides the well-known immunosuppressive-inducer antigens such as MUC1, can improve the efficacy of DC-based vaccines in the pro-inflammatory tumor microenvironment.

Several techniques have been developed to express the desired peptide-MHC complex in DCs via mRNAs, i.e., electroporation, lipid-mediated transfection, and mRNA co-incubation, which are reviewed elsewhere (140). In DC-based vaccines, the mRNA-based technique is grouped into single-antigen mRNA and total-antigens mRNA. Based on the heterogenicity of tumoral cells, the total-antigens mRNA might be a better option. Van Nuffel et al. have loaded the mRNAs of gp100, MAGE-A3, and MAGE-C2 into the DCs via the pST1-SIINFEKL vector. These modified DCs could induce substantial levels of functional CD4^+^ and CD8^+^ cells in melanoma patients (141). Since the peptides from loaded mRNAs must be coupled with the MHC compound to mount the anti-tumoral immune responses, the chimeric lysosome-associated membrane protein-1 (LAMP-1) with the tumoral antigen can facilitate this process (142). Consistent with this, Su et al. (143) have shown that the mRNA of tumoral antigen/LAMP-1 can enhance the peptide/MHC-I presentation and the anti-tumoral immune responses in affected patients.

#### siRNA

The first DC-based vaccine for cancer was approved in 2010 for prostate cancer patients (144). Based on a report, DC-based vaccines can only induce an 8.5% response rate (145). The reason for this low response rate can be attributable to the immunosuppressive tumor microenvironment (146). In the siRNA technique, the inhibitory axes can be targeted, which can pave the way for a better presentation of decoded tumoral antigens in DCs. As mentioned above, cDC1 can overexpress PD-L1 and suppress anti-tumoral immune responses (147). It has been reported that silencing PD-L1 in DC-based vaccines can remarkably increase the priming of CD8^+^ (148). Nanoparticles are another appealing vehicle to translocate siRNA to DCs. Hobo et al. have demonstrated that loading antigen mRNA with silencing PD-L1/PD-L2 via DLin-KC2-DMA-containing lipid nanoparticles can enhance the CD8^+^ stimulation. This approach has not affected the phenotype and migratory effect of DCs (149).

Another plausible target might be silencing IDO in DCs. Flatekval et al. have shown that loading IDO-siRNA can substantially improve the stimulation of T-cells (150). Moreover, the DC-loaded with IDO-siRNA can stimulate the CD8^+^ and decrease the Foxp3^+^ T-reg development in mice bearing breast cancer (151). In patients with gynecological cancers, the silencing IDO in DC vaccines has resulted in the up-regulation of C-C chemokine receptor type 7 (CR7), CD40, CD80, and CD86, which can mediate the DC migration to lymph nodes and provide the co-stimulatory axes to activate anti-tumoral immune responses (152). In melanoma-bearing mice, treatment with IDO-siRNA using mannosed liposomes has decreased apoptosis in CD8 and CD4 T-cells (153). Delivering IDO1-siRNA via multifunctional envelope-type nano device (MEND) containing a YSK12-C4 (YSK12-MEND) to DCs can reduce the Treg cells without affecting MHC-II. Therefore, nanotechnology might bring new opportunities to DC-based cancer immunotherapy (154).

## The Lessons we Have Learned and the Lessons we Should Learn

The following sections aim to collect the learned lessons from successful and unsuccessful clinical trials. Based on these lessons and the results of preclinical studies, we will present our proposed strategy for treating melanoma patients.

### Suppressing the MAPK and PI3K/AKT Signaling Pathway: Time to Rethink About It?

The two main oncogenic pathways of melanoma, i.e., the MAPK and PI3K/AKT pathways, were the center of efforts to overcome melanoma. However, chemotherapy against these pathways has not provided well-tolerable treatment for affected patients. Due to the proliferative and anti-apoptotic ability of the MAPK signaling pathway, inhibitors targeting this signaling pathway were developed; however, these inhibitors failed to fulfill the desired outcomes because of the complexity of melanoma pathways. Vemurafenib is the first generation of BRaf inhibitor, which has passed the phase I trial with an 81% response (155). In its phase II trial, the total response rate was 53%. However, the development of cutaneous squamous cell carcinoma along with other side effects in melanoma patients put the safety of this agent at risk (156). In phase III randomized trial of vemurafenib, vemurafenib administration reduced 63% the risk of death and decreased 74% the risk of death/disease advancement. This agent caused cutaneous squamous-cell carcinoma and alopecia in 12 and 8% of the patients, respectively (157). One of the concerning issues with BRaf inhibitors is the ever-increasing risk of developing resistance (158). Next, the administration BRaf inhibitors have been correlated with the development of cancers, e.g., cutaneous squamous cell carcinomas, leukemias, and colon cancer (158). Another proposed inhibitor of the MAPK pathway is Binimetinib, which can suppress MEK1/2 in patients with mutant NRas melanoma (159). In the phase II trial of binimetinib, binimetinib has failed to elicit the complete response in melanoma patients. This agent could exert partial response in 20% of the patients with NRas-mutated melanoma. The side effects of the agent were not negligible, e.g., irregular heart rate and developing acneiform dermatitis were seen in 60% of patients with mutant NRas (160).

The PI3K/AKT signaling pathway is another main pathway of melanoma development. Therefore, developing the PI3K/AKT pathway inhibitors was another appealing approach for treating melanoma patients. However, there have been serious issues with the efficacy of the PI3K/AKT pathway inhibitors. Although preclinical studies have demonstrated the desired outcomes in treating melanoma cells with CCI-779 mTOR kinase inhibitor, its phase II trial has revealed poor outcomes about the application of this agent (161). Perifosine, the AKT inhibitor, has failed to respond to any beneficial responses in melanoma patients (162). A possible explanation can be the overstimulation of the MAPK pathway in the NRas and negative feedback manner. Collectively, the MAPK pathway and PI3K/AKT are two main carcinogenic pathways in melanoma patients. Thus, inhibition of one of them can augment the other pathway, which leads to chemoresistance.

### RNA-Modified DC-Vaccines for MUC1 Might Be a Key Step in Cancer Therapy

Aberrant MUC1, which is substantially expressed in melanoma cells, has been implicated in the induction of immunosuppressive tumor microenvironment (10, 11). As discussed above, this tumor antigen is closely associated with the MAPK signaling pathway (Figure 2). This immunogenic antigen has a remarkable role in the recruitment of MDSCs and the release of IDO, which can lead to the inactivation of DCs (163). Moreover, MUC1 has been implicated in the up-regulation of PD-L1, which suppresses anti-tumoral immune responses. Therefore, this antigen has a focal role in melanoma development and suppressing anti-tumoral immune responses. There have been substantial advances in MUC1-targeting cancer vaccines, i.e., subunit vaccines, DNA-based vaccines, viral vector-based vaccines, glycopeptide vaccines, and DC-based vaccines. The detailed description of other types of vaccines, i.e., subunit vaccines, DNA-based vaccines, viral vector-based vaccines, glycopeptide vaccines, is reviewed elsewhere (82). Although DC-based vaccines are well-tolerable, their low response rate cannot lead to their clinical translation (145). Various studies have shown the undesirable response rate of traditional peptide-loaded DC vaccines, like MUC1 loaded-DC vaccines, compared to RNA-modified DC vaccines, especially in melanoma (see below).

Although MUC1 loaded-DC vaccines have shown beneficial outcomes for patients with non-small cell lung cancer (84), peptide-loaded DC-vaccines have failed to show any superiority over the administration of ipilimumab, CTLA-4 targeting antibody, in melanoma patients (164). In phase I clinical trial of MUC1 loaded DC-based vaccine, the vaccines have induced low-titer antibody in adenocarcinoma patients; however, no toxicity has been observed (165). In phase I/II clinical trial of MUC1-loaded DC-vaccines, DCs loaded with the MUC1 peptide have not induced toxicity in patients with pancreatic and biliary cancers. Following each administration of these DC-vaccines, there has been a transient increase in the level of CD8^+^ and CD4^+^ T-cells (166). In melanoma patients, the traditional loading of DCs with the peptide of tumor-associated antigen, Mage-3A1, has not induced sustained anti-tumoral immune responses (167). In patients with metastatic melanoma, the overall response of DC-vaccines, developed from MoDCs loaded with peptides of tumor antigens, has been disappointing (168). In phase Ib trial of tumor lysate DC vaccines, DC-vaccines, manufactured via co-culturing peripheral blood mononuclear cells with granulocyte macrophage-colony stimulating factor and IL-4 and loaded with tumor lysate/keyhole limpet hemocyanin, have not resulted in clinical response in patients with metastatic melanoma (169). In melanoma patients, the DC-based vaccines, loaded with wild-type/modified gp100 and tyrosinase peptides, have only had limited clinical benefits (170). However, the RNA-modified DC vaccine is a relatively novel and promising approach to stimulate anti-tumoral immune responses and develop memory cells. The combination of nanoparticle-delivered MUC1 mRNA DC-vaccine with anti-CTLA-4 monoclonal antibody has induced robust anti-tumoral immune responses in mice models bearing triple-negative breast cancer (80).

### Established Immune Checkpoint Inhibitors/Novel Immune Checkpoints Inhibitors for Targeting PD-L1/PD-1 Axis and CTLA-4 and Their Values in Combination Therapy

The immunosuppressive tumor microenvironment of melanoma inhibits the development of anti-tumoral immune responses. The overexpression of PD-L1 and CTL-A4 on the tumoral cells shields the tumoral cells from immune responses. Based on recent meta-analyses, there has been a remarkable association between tumor-infiltrating lymphocytes and tumoral PD-L1 expression in triple-negative breast cancer and melanoma (55, 171). Despite an increased level of tumor-infiltrating lymphocyte, the PD-L1 up-regulation in tumors represses anti-tumoral immune responses (172, 173). Upon the DC-based vaccine administration, notable upregulation of PD-L1 on the melanoma cells attenuates the CD8^+^ cell stimulation (174). Therefore, targeting these inhibitory axes can stimulate anti-tumoral immune responses. Following the recognition of the inhibitory role of PD-1, pembrolizumab and nivolumab developed. These monoclonal antibodies have shown better results than ipilimumab, a monoclonal antibody against CTLA-4, in terms of overall survival rate (175, 176). A meta-analysis by Jing L et al. has shown that the PD-1 inhibitors can considerably improve the progression-free survival and overall survival in melanoma patients (HR = 0.53 95%CI: 0.48–0.59 and HR = 0.60 95% CI: 0.53–0.69, respectively) (177). In another meta-analysis, the nivolumab/pembrolizumab vs. chemotherapy treatment has improved the progression-free survival in melanoma patients (HR = 0.42 95% CI: 0.36–0.49). The nivolumab with ipilimumab vs. ipilimumab, and nivolumab vs. ipilimumab treatment have substantially improved the progression-free survival in melanoma patients (HR = 0.41 95% CI: 0.30–0.52, and HR = 0.58 95% CI: 0.50–0.66). The reported toxicity was higher in the nivolumab with ipilimumab treatment; however, the symptoms were manageable (178).

Although most studies investigate the inhibition of PD-1 in cancer contexts, durvalumab and avelumab are novel agents, which can block tumoral PD-L1. The combination of tremelimumab, as an inhibitor of CTLA-4, and durvalumab have demonstrated promising results for patients with overexpressed PD-L1 solid tumors. However, the combination of durvalumab with tremelimumab needs a close follow-up because of the increased incidence of adverse events in treated patients (179). Although tumors with low expression of PD-L1 may not benefit from durvalumab the same as the tumors with overexpressed PD-L1, the combination of durvalumab and tremelimumab has shown desirable outcomes in patients with low PD-L1 expressed non-small cell lung cancer (180). In phase 1b clinical trial of avelumab, avelumab has resulted in a 19.9% objective response rate without causing death in patients with non-small cell lung cancer (181). In phase 1b clinical trial of avelumab, the avelumab administration has been associated with a 42.1% overall response rate without grade 4 treatment-related adverse events in patients with PD-L1^+^ melanoma (182). Thus, the safety and efficacy of these novel agents allow for further investigations.

Following the CTLA-4 and its inhibitory effect on the immune response, the first monoclonal antibody targeting CTLA-4 was introduced. However, ipilimumab was only beneficial on a small portion of patients with melanoma (175). In patients with advanced melanoma, pembrolizumab administration has resulted in better outcomes in terms of progression-free survival and subsequent toxicity than ipilimumab administration (183). In patients with metastatic cutaneous melanoma, pembrolizumab with ipilimumab resulted in a response rate of 38% with minor toxicity (184).

### Suppressing IDO and Its Value in Combination Therapy

Since IDO can also be expressed from tumoral cells (99, 100), the silencing of DC-derived IDO might not suffice to downregulate the produced IDO in the tumor microenvironment. Epacadostat is an oral inhibitor of the IDO1 enzyme. Preclinical findings have indicated that the treatment of DCs with epacadostat can increase the lysis activity of T-cells and decrease immature Tim3^+^ NK cells (185). Its combination with nivolumab has been well-tolerable and shown favorable clinical responses in patients with advanced melanoma (186). The combination of pembrolizumab with indoximod, an IDO inhibitor, has resulted in a 55.7% overall response rate in patients with advanced melanoma (187). In patients with metastatic melanoma, the combination of epacadostat with ipilimumab has been well-tolerable, and the clinical response has been considerable, especially in patients without prior immunotherapy (188). However, the combination of epacadostat and pembrolizumab has not shown superiority over the monotherapy with pembrolizumab in melanoma patients (189). A meta-analysis might be needed to answer these conflicting data regarding the epacadostat in melanoma patients. Furthermore, there is no clinical trial evaluating the efficacy of epacadostat with engineered DC-based vaccines and immune checkpoint inhibitors in melanoma patients.

### Clinical Trials for Treating Melanoma Patients

Table 1 aims to summarize the clinical trials regarding melanoma treatment. Although some preclinical investigations have demonstrated relatively promising outcomes, most of these listed clinical trials are in phase II. In this phase, the efficacy and safety of medication are determined. Most of the studies exclusively focus on one or two aspects of the biology of melanoma and the immune system. However, as discussed above, this kind of approach might not be promising for treating melanoma patients. The futility of traditional DC-based vaccine administration may stem from the inhibitory roles of IDO and immune checkpoints, e.g., PD-L1/PD-1 and CTLA-4 axes (Figure 2). the NCT02678741 clinical trial utilizes the tumor lysates for manufacturing DC-based vaccines, which might be challenging to induce remarkable anti-tumoral immune responses. No detailed information about the process of DC-based manufacturing in the NCT03092453 clinical trial has been provided. There is also no information about the completed NCT00125749 clinical trial.

**Table 1:** Some of the clinical trials were designed for patients with melanoma.

**Medication**	**Mechanism of action**	**Clinical trial phase**	**Study start date**	**The status**	**ClinicalTrials.gov identifier**
DC-vaccine	Biological anti-tumoral immunity	Phase II	2002	Terminated	NCT01042366
DC-vaccine	Biological anti-tumoral immunity	Phase I	2005	Completed	NCT00125749
Binimetinib and Encorafenib	Inhibition of the MAPK pathway and BRaf gene, respectively	Phase II	2020	Not yet recruiting	NCT04221438
Pembrolizumab and Ipilimumab	Inhibition of PD-1 axis and CTLA-4 axis	Phase II	2019	Active, not recruiting	NCT03873818
Nivolumab and Ipilimumab	Inhibition of PD-1 axis and CTLA-4 axis	Phase II	2016	Active, not recruiting	NCT02970981
Pembrolizumab, Cyclophosphamide, and DC-based vaccine	Inhibiting PD-1/PD-L1 axis, inhibiting protein synthesis, and Biological anti-tumoral immunity	Phase I	2017	Recruiting	NCT03092453
Standard of care immune checkpoint inhibitors and DC-based vaccine	Inhibiting immune checkpoints and inducing biological anti-tumoral immunity	Phase I/II	2016	Active, not recruiting	NCT02678741

*DC-vaccine, dendritic cell vaccine; PD-1: programmed cell death protein 1*.

## Concluding Remarks

Developing agents to suppress the oncogenic pathways had been the cornerstone of efforts to treat cancer patients. However, their side-effects and the heterogeneous nature of tumor cells have posed daunting challenges for chemotherapy. On the other hand, immunotherapy has been revolutionizing cancer therapy. Despite the low response rate of traditional peptide-based DC vaccines, the novel RNA-modified DC vaccines have shown low toxicity and promising results in preclinical studies. Loading coded mRNA for desired antigens and siRNA for targeting inhibitory axes might be a promising approach in developing DC vaccines. The data from the single-cell RNA-sequencing, along with the well-established tumoral antigens like MUC1, can help us design the desired mRNA in heterogeneous and ever-progressing cancers. The immunosuppressive tumor microenvironment of melanoma might owe to the fact that there are multiple auto-inductive loops between tumoral expressed immune checkpoints, oncogenic signaling pathways, IDO, and immunosuppressive cells. Indeed, combination therapy of immune checkpoint inhibitors and IDO might transform the immunosuppressive tumor microenvironment into the pro-inflammatory tumor microenvironment. The target antigens for gene-modified DC vaccines can be provided from the data of single-cell RNA-sequencing and previous well-known pro-tumoral tumor antigens, e.g., MUC1. Thus, this combination therapy and the administration of RNA-modified DC vaccines can suppress tumor development and provide long-lasting immunity against tumor antigens.

## Author Contributions

MS: the first author of the manuscript, collected the data, and wrote the primary version of the manuscript. KH, AD, and NS: contributed to English editing and revision of the manuscript, and also helped with data categorization. BB and VR: the corresponding authors of the manuscript contributed to writing the main text of the manuscript and supervised the manuscript. All authors contributed to the article and approved the submitted version.

## Conflict of Interest

The authors declare that the research was conducted in the absence of any commercial or financial relationships that could be construed as a potential conflict of interest.

## References

[1] Gray-Schopfer V Wellbrock C Marais R. Melanoma biology and new targeted therapy. Nature. (2007) 445:851. 10.1038/nature05661 17314971

[2] Carvajal RD Kaushal M Kehrer MD Domary S Brady Md. Melanoma and Other Skin Cancers (2015).

[3] Matthews NH Li W-Q Qureshi AA Weinstock MA Cho E. Epidemiology of melanoma. In: Ward WH Farma JM editors. Cutaneous Melanoma: Etiology and Therapy. Brisbane, QLD: Codon Publications (2017) 3–22. 29461782

[4] Kumar V Abbas AK Fausto N Aster JC. Robbins and Cotran Pathologic Basis of Disease, Professional Edition E-Book. Elsevier Health Sciences (2014).

[5] Leone P Berardi S Frassanito MA Ria R De Re V Cicco S. Dendritic cells accumulate in the bone marrow of myeloma patients where they protect tumor plasma cells from CD8+ T-cell killing. Blood. (2015) 126:1443–51. 10.1182/blood-2015-01-623975 26185130 PMC4592278

[6] Racanelli V Behrens S-E Aliberti J Rehermann B. Dendritic cells transfected with cytopathic self-replicating RNA induce crosspriming of CD8+ T cells and antiviral immunity. Immunity. (2004) 20:47–58. 10.1016/S1074-7613(03)00353-4 14738764

[7] Gutiérrez-Martínez E Planès R Anselmi G Reynolds M Menezes S Adiko AC. Cross-presentation of cell-associated antigens by MHC class I in dendritic cell subsets. Front. Immunol. (2015) 6:363. 10.3389/fimmu.2015.00363 26236315 PMC4505393

[8] Berraondo P Minute L Ajona D Corrales L Melero I Pio R. Innate immune mediators in cancer: between defense and resistance. Immunol. Rev. (2016) 274:290–306. 10.1111/imr.12464 27782320

[9] Leone P Shin E-C Perosa F Vacca A Dammacco F Racanelli V. MHC class I antigen processing and presenting machinery: organization, function, and defects in tumor cells. J. Natl. Cancer Inst. (2013) 105:1172–87. 10.1093/jnci/djt184 23852952

[10] Wang X Lan H Li J Su Y Xu L. Muc1 promotes migration and lung metastasis of melanoma cells. Am. J. Cancer Res. (2015) 5:2590. 26609470 PMC4633892

[11] Shadbad MA Hajiasgharzadeh K Baradaran B. Cross-talk between myeloid-derived suppressor cells and Mucin1 in breast cancer vaccination: on the verge of a breakthrough. Life Sci. (2020) 258:118128. 10.1016/j.lfs.2020.118128 32710947

[12] Dhomen N Marais R. BRAF signaling and targeted therapies in melanoma. Hematol. Oncol. Clin. (2009) 23:529–45. 10.1016/j.hoc.2009.04.001 19464601

[13] Sharma A Sharma AK Madhunapantula SV Desai D Huh SJ Mosca P. Targeting Akt3 signaling in malignant melanoma using isoselenocyanates. Clin. Cancer Res. (2009) 15:1674–85. 10.1158/1078-0432.CCR-08-2214 19208796 PMC2766355

[14] Giehl K. Oncogenic Ras in tumour progression and metastasis. Biol. Chem. (2005) 386:193–205. 10.1515/BC.2005.025 15843165

[15] Kohno M Pouyssegur J. Targeting the ERK signaling pathway in cancer therapy. Ann. Med. (2006) 38:200–11. 10.1080/07853890600551037 16720434

[16] Gonzalez D Fearfield L Nathan P Tanière P Wallace A Brown E. BRAF mutation testing algorithm for vemurafenib treatment in melanoma: recommendations from an expert panel. Br. J. Dermatol. (2013) 168:700–7. 10.1111/bjd.12248 23360189

[17] Dankort D Curley DP Cartlidge RA Nelson B Karnezis AN Damsky Jr WE. Braf V600E cooperates with Pten loss to induce metastatic melanoma. Nat. Genet. (2009) 41:544. 10.1038/ng.356 19282848 PMC2705918

[18] Dhomen N Reis-Filho JS Da Rocha Dias S Hayward R Savage K Delmas V. Oncogenic braf induces melanocyte senescence and melanoma in mice. Cancer Cell. (2009) 15:294–303. 10.1016/j.ccr.2009.02.022 19345328

[19] Hayward NK Wilmott JS Waddell N Johansson PA Field MA Nones K. Whole-genome landscapes of major melanoma subtypes. Nature. (2017) 545:175–80. 10.1038/nature22071 28467829

[20] Michailidou C Jones M Walker P Kamarashev J Kelly A Hurlstone AF. Dissecting the roles of Raf-and PI3K-signalling pathways in melanoma formation and progression in a zebrafish model. Dis. Models Mech. (2009) 2:399–411. 10.1242/dmm.001149 19470611

[21] Fernandez PC Frank SR Wang L Schroeder M Liu S Greene J. Genomic targets of the human c-Myc protein. Genes Dev. (2003) 17:1115–29. 10.1101/gad.1067003 12695333 PMC196049

[22] Lin X Sun R Zhao X Zhu D Zhao X Gu Q. C-myc overexpression drives melanoma metastasis by promoting vasculogenic mimicry via c-myc/snail/Bax signaling. J. Mol. Med. (2017) 95:53–67. 10.1007/s00109-016-1452-x 27543492

[23] Cao Z Bao M Miele L Sarkar FH Wang Z Zhou Q. Tumour vasculogenic mimicry is associated with poor prognosis of human cancer patients: a systemic review and meta-analysis. Eur. J. Cancer. (2013) 49:3914–23. 10.1016/j.ejca.2013.07.148 23992642

[24] Zhuang D Mannava S Grachtchouk V Tang W Patil S Wawrzyniak J. C-MYC overexpression is required for continuous suppression of oncogene-induced senescence in melanoma cells. Oncogene. (2008) 27:6623–34. 10.1038/onc.2008.258 18679422 PMC3808965

[25] Shain AH Yeh I Kovalyshyn I Sriharan A Talevich E Gagnon A. The genetic evolution of melanoma from precursor lesions. N. Engl. J. Med. (2015) 373:1926–36. 10.1056/NEJMoa1502583 26559571

[26] Ozaki T Nakagawara A. Role of p53 in cell death and human cancers. Cancers. (2011) 3:994–1013. 10.3390/cancers3010994 24212651 PMC3756401

[27] Du W Searle JS. The rb pathway and cancer therapeutics. Curr. Drug Targets. (2009) 10:581–9. 10.2174/138945009788680392 19601762 PMC3151466

[28] Cen L Carlson BL Schroeder MA Ostrem JL Kitange GJ Mladek AC. p16-Cdk4-Rb axis controls sensitivity to a cyclin-dependent kinase inhibitor PD0332991 in glioblastoma xenograft cells. Neuro-oncology. (2012) 14:870–81. 10.1093/neuonc/nos114 22711607 PMC3379801

[29] Kucharska A Rushworth LK Staples C Morrice NA Keyse SM. Regulation of the inducible nuclear dual-specificity phosphatase DUSP5 by ERK MAPK. Cell. Signal. (2009) 21:1794–805. 10.1016/j.cellsig.2009.07.015 19666109

[30] Brunet A Roux D Lenormand P Dowd S Keyse S Pouysségur J. Nuclear translocation of p42/p44 mitogen-activated protein kinase is required for growth factor-induced gene expression and cell cycle entry. EMBO J. (1999) 18:664–74. 10.1093/emboj/18.3.664 9927426 PMC1171159

[31] Burch PM Yuan Z Loonen A Heintz NH. An extracellular signal-regulated kinase 1-and 2-dependent program of chromatin trafficking of c-Fos and Fra-1 is required for cyclin D1 expression during cell cycle reentry. Mol. Cell. Biol. (2004) 24:4696–709. 10.1128/MCB.24.11.4696-4709.2004 15143165 PMC416393

[32] Marampon F Ciccarelli C Zani BM. Down-regulation of c-Myc following MEK/ERK inhibition halts the expression of malignant phenotype in rhabdomyosarcoma and in non muscle-derived human tumors. Mol. Cancer. (2006) 5:31. 10.1186/1476-4598-5-31 16899113 PMC1560159

[33] Walsh S Margolis SS Kornbluth S. Phosphorylation of the Cyclin B1 cytoplasmic retention sequence by mitogen-activated protein kinase and Plx1 1 NIH RO1 GM60500 to SKSK is a scholar of the leukemia and lymphoma society. Mol. Cancer Res. (2003) 1:280–9. 12612056

[34] Allan LA Morrice N Brady S Magee G Pathak S Clarke PR. Inhibition of caspase-9 through phosphorylation at Thr 125 by ERK MAPK. Nature Cell Biol. (2003) 5:647–54. 10.1038/ncb1005 12792650

[35] Harada H Grant S. Targeting the regulatory machinery of BIM for cancer therapy. Crit. Rev. Eukaryot. Gene Expr. (2012) 22:117–29. 10.1615/CritRevEukarGeneExpr.v22.i2.40 22856430 PMC3834587

[36] Ries S Biederer C Woods D Shifman O Shirasawa S Sasazuki T. Opposing effects of Ras on p53: transcriptional activation of mdm2 and induction of p19ARF. Cell. (2000) 103:321–30. 10.1016/S0092-8674(00)00123-9 11057904

[37] Hocker TL Singh MK Tsao H. Melanoma genetics and therapeutic approaches in the 21st century: moving from the benchside to the bedside. J. Invest. Dermatol. (2008) 128:2575–95. 10.1038/jid.2008.226 18927540

[38] Slipicevic A Holm R Nguyen MT Bøhler PJ Davidson B Flørenes VA. Expression of activated Akt and PTEN in malignant melanomas: relationship with clinical outcome. Am. J. Clin. Pathol. (2005) 124:528–36. 10.1309/YT58WWMTA6YR1PRV 16146807

[39] Ogawara Y Kishishita S Obata T Isazawa Y Suzuki T Tanaka K. Akt enhances Mdm2-mediated ubiquitination and degradation of p53. J. Biol. Chem. (2002) 277:21843–50. 10.1074/jbc.M109745200 11923280

[40] Mitsuuchi Y Johnson SW Selvakumaran M Williams SJ Hamilton TC Testa JR. The phosphatidylinositol 3-kinase/AKT signal transduction pathway plays a critical role in the expression of p21WAF1/CIP1/SDI1 induced by cisplatin and paclitaxel. Cancer Res. (2000) 60:5390–4. 11034077

[41] Majumder PK Febbo PG Bikoff R Berger R Xue Q Mcmahon LM. mTOR inhibition reverses Akt-dependent prostate intraepithelial neoplasia through regulation of apoptotic and HIF-1-dependent pathways. Nat. Med. (2004) 10:594–601. 10.1038/nm1052 15156201

[42] Düvel K Yecies JL Menon S Raman P Lipovsky AI Souza AL. Activation of a metabolic gene regulatory network downstream of mTOR complex 1. Mol. Cell. (2010) 39:171–83. 10.1016/j.molcel.2010.06.022 20670887 PMC2946786

[43] Neufeld TP. TOR-dependent control of autophagy: biting the hand that feeds. Curr. Opin. Cell Biol. (2010) 22:157–68. 10.1016/j.ceb.2009.11.005 20006481 PMC2854204

[44] Lorente J Velandia C Leal JA Garcia-Mayea Y Lyakhovich A Kondoh H. The interplay between autophagy and tumorigenesis: exploiting autophagy as a means of anticancer therapy. Biol. Rev. (2018) 93:152–65. 10.1111/brv.12337 28464404

[45] Mayer C Grummt I. Ribosome biogenesis and cell growth: mTOR coordinates transcription by all three classes of nuclear RNA polymerases. Oncogene. (2006) 25:6384–91. 10.1038/sj.onc.1209883 17041624

[46] Nandagopal N Roux PP. Regulation of global and specific mRNA translation by the mTOR signaling pathway. Translation. (2015) 3:e983402. 10.4161/21690731.2014.983402 26779414 PMC4682803

[47] Hosseinkhani N Derakhshani A Kooshkaki O Abdoli Shadbad M Hajiasgharzadeh K Baghbanzadeh A. Immune checkpoints and CAR-T Cells: the pioneers in future cancer therapies? Int. J. Mol. Sci. (2020) 21:8305. 10.3390/ijms21218305 33167514 PMC7663909

[48] Payandeh Z Khalili S Somi MH Mard-Soltani M Baghbanzadeh A Hajiasgharzadeh K. PD-1/PD-L1-dependent immune response in colorectal cancer. J. Cell. Physiol. (2020) 235:5461–75. 10.1002/jcp.29494 31960962

[49] Massi D Brusa D Merelli B Falcone C Xue G Carobbio A. The status of PD-L1 and tumor-infiltrating immune cells predict resistance and poor prognosis in BRAFi-treated melanoma patients harboring mutant BRAFV600. Ann. Oncol. (2015) 26:1980–7. 10.1093/annonc/mdv255 26037795

[50] Kleffel S Posch C Barthel SR Mueller H Schlapbach C Guenova E. Melanoma cell-intrinsic PD-1 receptor functions promote tumor growth. Cell. (2015) 162:1242–56. 10.1016/j.cell.2015.08.052 26359984 PMC4700833

[51] Mayoux M Roller A Pulko V Sammicheli S Chen S Sum E. Dendritic cells dictate responses to PD-L1 blockade cancer immunotherapy. Sci. Transl. Med. (2020) 12:eaav7431. 10.1126/scitranslmed.aav7431 32161104

[52] Kwak G Kim D Nam GH Wang SY Kim IS Kim SH . Programmed cell death protein Ligand-1 silencing with polyethylenimine-dermatan sulfate complex for dual inhibition of melanoma growth. ACS Nano. (2017) 11:10135–46. 10.1021/acsnano.7b04717 28985469 PMC5697980

[53] Yang J Dong M Shui Y Zhang Y Zhang Z Mi Y. A pooled analysis of the prognostic value of PD-L1 in melanoma: evidence from 1062 patients. Cancer Cell Int. (2020) 20:1–11. 10.1186/s12935-020-01187-x 32256205 PMC7106672

[54] Zheng F Dang J Zha H Zhang B Lin M Cheng F. PD-L1 promotes self-renewal and tumorigenicity of malignant melanoma initiating cells. BioMed Res. Int. (2017) 2017:1293201. 10.1155/2017/1293201 29250533 PMC5700500

[55] Xu J Wang F Yan Y Zhang Y Du Y Sun G. Prognostic and clinicopathological value of PD-L1 in melanoma: a meta-analysis. Am. J. Med. Sci. (2020) 359:339–46. 10.1016/j.amjms.2020.03.020 32498941

[56] Atefi M Avramis E Lassen A Wong DJ Robert L Foulad D. Effects of MAPK and PI3K pathways on PD-L1 expression in melanoma. Clin. Cancer Res. (2014) 20:3446–57. 10.1158/1078-0432.CCR-13-2797 24812408 PMC4079734

[57] Jiang X Zhou J Giobbie-Hurder A Wargo J Hodi FS. The activation of MAPK in melanoma cells resistant to BRAF inhibition promotes PD-L1 expression that is reversible by MEK and PI3K inhibition. Clin. Cancer Res. (2013) 19:598–609. 10.1158/1078-0432.CCR-12-2731 23095323

[58] Kakavand H Wilmott JS Menzies AM Vilain R Haydu LE Yearley JH. PD-L1 expression and tumor-infiltrating lymphocytes define different subsets of MAPK inhibitor–treated melanoma patients. Clin. Cancer Res. (2015) 21:3140–8. 10.1158/1078-0432.CCR-14-2023 25609064

[59] Sumimoto H Imabayashi F Iwata T Kawakami Y. The BRAF–MAPK signaling pathway is essential for cancer-immune evasion in human melanoma cells. J. Exp. Med. (2006) 203:1651–6. 10.1084/jem.20051848 16801397 PMC2118331

[60] Rodić N Anders RA Eshleman JR Lin M-T Xu H Kim JH. PD-L1 expression in melanocytic lesions does not correlate with the BRAF V600E mutation. Cancer Immunol. Res. (2015) 3:110–5. 10.1158/2326-6066.CIR-14-0145 25370533 PMC4324161

[61] Frederick DT Piris A Cogdill AP Cooper ZA Lezcano C Ferrone CR. BRAF inhibition is associated with enhanced melanoma antigen expression and a more favorable tumor microenvironment in patients with metastatic melanoma. Clin. Cancer Res. (2013) 19:1225–31. 10.1158/1078-0432.CCR-12-1630 23307859 PMC3752683

[62] Stutvoet TS Kol A De Vries EG De Bruyn M Fehrmann RS Terwisscha Van Scheltinga AG. MAPK pathway activity plays a key role in PD-L1 expression of lung adenocarcinoma cells. J. Pathol. (2019) 249:52–64. 10.1002/path.5280 30972766 PMC6767771

[63] Della Corte CM Barra G Ciaramella V Di Liello R Vicidomini G Zappavigna S. Antitumor activity of dual blockade of PD-L1 and MEK in NSCLC patients derived three-dimensional spheroid cultures. J. Exp. Clin. Cancer Res. (2019) 38:253. 10.1186/s13046-019-1257-1 31196138 PMC6567578

[64] Loi S Dushyanthen S Beavis PA Salgado R Denkert C Savas P. RAS/MAPK activation is associated with reduced tumor-infiltrating lymphocytes in triple-negative breast cancer: therapeutic cooperation between MEK and PD-1/PD-L1 immune checkpoint inhibitors. Clin. Cancer Res. (2016) 22:1499–509. 10.1158/1078-0432.CCR-15-1125 26515496 PMC4794351

[65] Parsa AT Waldron JS Panner A Crane CA Parney IF Barry JJ. Loss of tumor suppressor PTEN function increases B7-H1 expression and immunoresistance in glioma. Nat. Med. (2007) 13:84–8. 10.1038/nm1517 17159987

[66] Yang L Huang F Mei J Wang X Zhang Q Wang H. Posttranscriptional control of PD-L1 expression by 17β-estradiol via PI3K/Akt signaling pathway in ERα-positive cancer cell lines. Int. J. Gynecol. Cancer. (2017) 27:196–205. 10.1097/IGC.0000000000000875 27870715 PMC5258765

[67] Dong Y Richards J-A Gupta R Aung PP Emley A Kluger Y. PTEN functions as a melanoma tumor suppressor by promoting host immune response. Oncogene. (2014) 33:4632–42. 10.1038/onc.2013.409 24141770

[68] Liu S Chen S Yuan W Wang H Chen K Li D. PD-1/PD-L1 interaction up-regulates MDR1/P-gp expression in breast cancer cells via PI3K/AKT and MAPK/ERK pathways. Oncotarget. (2017) 8:99901. 10.18632/oncotarget.21914 29245948 PMC5725139

[69] Lan G Li J Wen Q Lin L Chen L Chen L. Cytotoxic T lymphocyte associated antigen 4 expression predicts poor prognosis in luminal B HER2-negative breast cancer. Oncol. Lett. (2018) 15:5093–7. 10.3892/ol.2018.7991 29552143 PMC5840684

[70] Mo X Zhang H Preston S Martin K Zhou B Vadalia N. Interferon-γ signaling in melanocytes and melanoma cells regulates expression of CTLA-4. Cancer Res. (2018) 78:436–50. 10.1158/0008-5472.CAN-17-1615 29150430 PMC5771950

[71] Stamper CC Zhang Y Tobin JF Erbe DV Ikemizu S Davis SJ. Crystal structure of the B7-1/CTLA-4 complex that inhibits human immune responses. Nature. (2001) 410:608–11. 10.1038/35069118 11279502

[72] Brunner MC Chambers CA Chan FK-M Hanke J Winoto A Allison JP. CTLA-4-mediated inhibition of early events of T cell proliferation. J. Immunol. (1999) 162:5813–20. 10229815

[73] Grohmann U Orabona C Fallarino F Vacca C Calcinaro F Falorni A. CTLA-4–Ig regulates tryptophan catabolism *in vivo*. Nat. Immunol. (2002) 3:1097–101. 10.1038/ni846 12368911

[74] Linsley PS Bradshaw J Greene J Peach R Bennett KL Mittler RS. Intracellular trafficking of CTLA-4 and focal localization towards sites of TCR engagement. Immunity. (1996) 4:535–43. 10.1016/S1074-7613(00)80480-X 8673700

[75] Chakravarti N Ivan D Trinh VA Glitza IC Curry JL Torres-Cabala C. High cytotoxic T-lymphocyte-associated antigen 4 and phospho-Akt expression in tumor samples predicts poor clinical outcomes in ipilimumab-treated melanoma patients. Melanoma Res. (2017) 27:24–31. 10.1097/CMR.0000000000000305 27768639

[76] Zhang B Dang J Ba D Wang C Han J Zheng F. Potential function of CTLA-4 in the tumourigenic capacity of melanoma stem cells. Oncol. Lett. (2018) 16:6163–70. 10.3892/ol.2018.9354 30344757 PMC6176363

[77] Peng M Mo Y Wang Y Wu P Zhang Y Xiong F. Neoantigen vaccine: an emerging tumor immunotherapy. Mol. Cancer. (2019) 18:1–14. 10.1186/s12943-019-1055-6 31443694 PMC6708248

[78] Rittig SM Haentschel M Weimer KJ Heine A Muller MR Brugger W. Intradermal vaccinations with RNA coding for TAA generate CD8+ and CD4+ immune responses and induce clinical benefit in vaccinated patients. Mol. Ther. (2011) 19:990–9. 10.1038/mt.2010.289 21189474 PMC3098631

[79] Roulois D Grégoire M Fonteneau J-F. MUC1-specific cytotoxic T lymphocytes in cancer therapy: induction and challenge. BioMed Res. Int. (2013) 2013:871936. 10.1155/2013/871936 23509794 PMC3591236

[80] Liu L Wang Y Miao L Liu Q Musetti S Li J. Combination immunotherapy of MUC1 mRNA nano-vaccine and CTLA-4 blockade effectively inhibits growth of triple negative breast cancer. Mol. Ther. (2018) 26:45–55. 10.1016/j.ymthe.2017.10.020 29258739 PMC5763160

[81] Papachristofilou A Hipp MM Klinkhardt U Früh M Sebastian M Weiss C. Phase Ib evaluation of a self-adjuvanted protamine formulated mRNA-based active cancer immunotherapy, BI1361849 (CV9202), combined with local radiation treatment in patients with stage IV non-small cell lung cancer. J. Immunother. Cancer. (2019) 7:38. 10.1186/s40425-019-0520-5 30736848 PMC6368815

[82] Gao T Cen Q Lei H. A review on development of MUC1-based cancer vaccine. Biomed. Pharmacother. (2020) 132:110888. 10.1016/j.biopha.2020.110888 33113416

[83] Scheikl-Gatard T Tosch C Lemonnier F Rooke R. Identification of new MUC1 epitopes using HLA-transgenic animals: implication for immunomonitoring. J. Transl. Med. (2017) 15:154. 10.1186/s12967-017-1254-0 28679396 PMC5499006

[84] Teramoto K Ozaki Y Hanaoka J Sawai S Tezuka N Fujino S. Predictive biomarkers and effectiveness of MUC1-targeted dendritic-cell-based vaccine in patients with refractory non-small cell lung cancer. Ther. Adv. Med. Oncol. (2017) 9:147–57. 10.1177/1758834016678375 28344660 PMC5349424

[85] Mcauley JL Linden SK Png CW King RM Pennington HL Gendler SJ. MUC1 cell surface mucin is a critical element of the mucosal barrier to infection. J. Clin. Invest. (2007) 117:2313–24. 10.1172/JCI26705 17641781 PMC1913485

[86] Chakraborty S Bonthu N Swanson BJ Batra SK. Role of mucins in the skin during benign and malignant conditions. Cancer Lett. (2011) 301:127–41. 10.1016/j.canlet.2010.11.004 21146919 PMC3232046

[87] Wang F Li Q Ni W Fang F Sun X Xie F. Expression of human full-length MUC1 inhibits the proliferation and migration of a B16 mouse melanoma cell line. Oncol. Rep. (2013) 30:260–8. 10.3892/or.2013.2440 23633115

[88] Deng M Jing DD Meng XJ. Effect of MUC1 siRNA on drug resistance of gastric cancer cells to trastuzumab. Asian Pac. J. Cancer Prev. (2013) 14:127–31. 10.7314/APJCP.2013.14.1.127 23534710

[89] Jin W Liao X Lv Y Pang Z Wang Y Li Q. MUC1 induces acquired chemoresistance by upregulating ABCB1 in EGFR-dependent manner. Cell Death Dis. (2017) 8:e2980. 10.1038/cddis.2017.378 28796259 PMC5596566

[90] Meerzaman D Shapiro PS Kim KC. Involvement of the MAP kinase ERK2 in MUC1 mucin signaling. Am. J. Physiol. Lung Cell. Mol. Physiol. (2001) 281:L86–91. 10.1152/ajplung.2001.281.1.L86 11404250

[91] Maeda T Hiraki M Jin C Rajabi H Tagde A Alam M. MUC1-C induces PD-L1 and immune evasion in triple-negative breast cancer. Cancer Res. (2018) 78:205–15. 10.1158/0008-5472.CAN-17-1636 29263152 PMC5754244

[92] Bouillez A Rajabi H Jin C Samur M Tagde A Alam M. MUC1-C integrates PD-L1 induction with repression of immune effectors in non-small-cell lung cancer. Oncogene. (2017) 36:4037–46. 10.1038/onc.2017.47 28288138 PMC5509481

[93] Li X Wang L Nunes D Troxler R Offner G. Pro-inflammatory cytokines up-regulate MUC1 gene expression in oral epithelial cells. J. Dent. Res. (2003) 82:883–7. 10.1177/154405910308201107 14578499

[94] Li Y-Y Hsieh L-L Tang R-P Liao S-K Yeh K-Y. Macrophage-derived interleukin-6 up-regulates MUC1, but down-regulates MUC2 expression in the human colon cancer HT-29 cell line. Cell. Immunol. (2009) 256:19–26. 10.1016/j.cellimm.2009.01.001 19201396

[95] Hornyák L Dobos N Koncz G Karányi Z Páll D Szabó Z. The role of indoleamine-2, 3-dioxygenase in cancer development, diagnostics, and therapy. Front. Immunol. (2018) 9:151. 10.3389/fimmu.2018.00151 29445380 PMC5797779

[96] Munn DH Sharma MD Hou D Baban B Lee JR Antonia SJ. Expression of indoleamine 2, 3-dioxygenase by plasmacytoid dendritic cells in tumor-draining lymph nodes. J. Clin. Invest. (2004) 114:280–90. 10.1172/JCI21583 15254595 PMC449750

[97] Lee JR Dalton RR Messina JL Sharma MD Smith DM Burgess RE. Pattern of recruitment of immunoregulatory antigen-presenting cells in malignant melanoma. Lab. Invest. (2003) 83:1457–66. 10.1097/01.LAB.0000090158.68852.D1 14563947

[98] Munn DH Mellor AL. Indoleamine 2, 3 dioxygenase and metabolic control of immune responses. Trends immunol. (2013) 34:137–43. 10.1016/j.it.2012.10.001 23103127 PMC3594632

[99] Shang K Wang Z Hu Y Huang Y Yuan K Yu Y. Gene silencing of indoleamine 2, 3-dioxygenase 1 inhibits lung cancer growth by suppressing T-cell exhaustion. Oncol. Lett. (2020) 19:3827–38. 10.3892/ol.2020.11477 32382333 PMC7202272

[100] Gide TN Allanson BM Menzies AM Ferguson PM Madore J Saw RP. Inter-and intrapatient heterogeneity of indoleamine 2, 3-dioxygenase expression in primary and metastatic melanoma cells and the tumour microenvironment. Histopathology. (2019) 74:817–28. 10.1111/his.13814 30589949

[101] Li H Bullock K Gurjao C Braun D Shukla SA Bossé D. Metabolomic adaptations and correlates of survival to immune checkpoint blockade. Nat. Commun. (2019) 10:1–6. 10.1038/s41467-019-12361-9 31554815 PMC6761178

[102] Speeckaert R Vermaelen K Van Geel N Autier P Lambert J Haspeslagh M. Indoleamine 2, 3-dioxygenase, a new prognostic marker in sentinel lymph nodes of melanoma patients. Eur. J. Cancer. (2012) 48:2004–11. 10.1016/j.ejca.2011.09.007 22033321

[103] Chevolet I Speeckaert R Haspeslagh M Neyns B Krüse V Schreuer M. Peritumoral indoleamine 2, 3-dioxygenase expression in melanoma: an early marker of resistance to immune control? Br. J. Dermatol. (2014) 171:987–95. 10.1111/bjd.13100 24814041

[104] Iga N Otsuka A Hirata M Kataoka TR Irie H Nakashima C. Variable indoleamine 2, 3-dioxygenase expression in acral/mucosal melanoma and its possible link to immunotherapy. Cancer Sci. (2019) 110:3434. 10.1111/cas.14195 31509303 PMC6824999

[105] Borcoman E De La Rochere P Richer W Vacher S Chemlali W Krucker C. Inhibition of PI3K pathway increases immune infiltrate in muscle-invasive bladder cancer. Oncoimmunology. (2019) 8:e1581556. 10.1080/2162402X.2019.1581556 31069145 PMC6492984

[106] Davis RJ Moore EC Clavijo PE Friedman J Cash H Chen Z. Anti-PD-L1 efficacy can be enhanced by inhibition of myeloid-derived suppressor cells with a selective inhibitor of PI3Kδ/γ. Cancer Res. (2017) 77:2607–19. 10.1158/0008-5472.CAN-16-2534 28364000 PMC5466078

[107] Ruhland MK Roberts EW Cai E Mujal AM Marchuk K Beppler C. Visualizing synaptic transfer of tumor antigens among dendritic cells. Cancer Cell. (2020) 37:786–799.e785. 10.1016/j.ccell.2020.05.002 32516589 PMC7671443

[108] Chen W Liang X Peterson AJ Munn DH Blazar BR. The indoleamine 2, 3-dioxygenase pathway is essential for human plasmacytoid dendritic cell-induced adaptive T regulatory cell generation. J. Immunol. (2008) 181:5396–404. 10.4049/jimmunol.181.8.5396 18832696 PMC2614675

[109] Segura E Amigorena S. Inflammatory dendritic cells in mice and humans. Trends Immunol. (2013) 34:440–5. 10.1016/j.it.2013.06.001 23831267

[110] Mu C Zhang G Huang J Qi B. pDC induced Treg proliferation through PD-1/PD-L1 signal and promote tumor immune escape of lung cancer with MPE. Eur. Respir. J. 44(Suppl 58).

[111] Ray A Das D Song Y Richardson P Munshi N Chauhan D. Targeting PD1–PDL1 immune checkpoint in plasmacytoid dendritic cell interactions with T cells, natural killer cells and multiple myeloma cells. Leukemia. (2015) 29:1441–4. 10.1038/leu.2015.11 25634684 PMC5703039

[112] Bruchhage KL Heinrichs S Wollenberg B Pries R. IL-10 in the microenvironment of HNSCC inhibits the CpG ODN-induced IFN-α secretion of pDCs. Oncol. Lett. (2018) 15:3985–90. 10.3892/ol.2018.7772 29456743 PMC5795883

[113] Pedroza-Gonzalez A Zhou G Vargas-Mendez E Boor PP Mancham S Verhoef C. Tumor-infiltrating plasmacytoid dendritic cells promote immunosuppression by Tr1 cells in human liver tumors. Oncoimmunology. (2015) 4:e1008355. 10.1080/2162402X.2015.1008355 26155417 PMC4485712

[114] Hildner K Edelson BT Purtha WE Diamond M Matsushita H Kohyama M. Batf3 deficiency reveals a critical role for CD8α+ dendritic cells in cytotoxic T cell immunity. Sci. (2008) 322:1097–100. 10.1126/science.1164206 19008445 PMC2756611

[115] Mittal D Vijayan D Putz EM Aguilera AR Markey KA Straube J. Interleukin-12 from CD103+ Batf3-dependent dendritic cells required for NK-cell suppression of metastasis. Cancer Immunol. Res. (2017) 5:1098–108. 10.1158/2326-6066.CIR-17-0341 29070650

[116] Böttcher JP Bonavita E Chakravarty P Blees H Cabeza-Cabrerizo M Sammicheli S. NK cells stimulate recruitment of cDC1 into the tumor microenvironment promoting cancer immune control. Cell. (2018) 172:1022–37.e1014. 10.1016/j.cell.2018.01.004 29429633 PMC5847168

[117] Zelenay S Van Der Veen AG Böttcher JP Snelgrove KJ Rogers N Acton SE. Cyclooxygenase-dependent tumor growth through evasion of immunity. Cell. (2015) 162:1257–70. 10.1016/j.cell.2015.08.015 26343581 PMC4597191

[118] Barry KC Hsu J Broz ML Cueto FJ Binnewies M Combes AJ. A natural killer–dendritic cell axis defines checkpoint therapy–responsive tumor microenvironments. Nat. Med. (2018) 24:1178–91. 10.1038/s41591-018-0085-8 29942093 PMC6475503

[119] Desch AN Randolph GJ Murphy K Gautier EL Kedl RM Lahoud MH. CD103+ pulmonary dendritic cells preferentially acquire and present apoptotic cell–associated antigen. J. Exp. Med. (2011) 208:1789–97. 10.1084/jem.20110538 21859845 PMC3171085

[120] Ferris ST Durai V Wu R Theisen DJ Ward JP Bern MD. cDC1 prime and are licensed by CD4+ T cells to induce anti-tumour immunity. Nature. (2020) 584:624–9. 10.1038/s41586-020-2611-3 32788723 PMC7469755

[121] Kobie JJ Wu RS Kurt RA Lou S Adelman MK Whitesell LJ. Transforming growth factor β inhibits the antigen-presenting functions and antitumor activity of dendritic cell vaccines. Cancer Res. (2003) 63:1860–4. 12702574

[122] Derakhshani A Silvestris N Hemmat N Asadzadeh Z Abdoli Shadbad M Nourbakhsh NS. Targeting TGF-β-mediated SMAD signaling pathway via novel recombinant cytotoxin II: a potent protein from naja naja oxiana venom in melanoma. Molecules. (2020) 25:5148. 10.3390/molecules25215148 33167431 PMC7663949

[123] Chrisikos TT Zhou Y Li HS Babcock RL Wan X Patel B. STAT3 inhibits CD103+ cDC1 vaccine efficacy in murine breast cancer. Cancers. (2020) 12:128. 10.3390/cancers12010128 31947933 PMC7017236

[124] Peng Q Qiu X Zhang Z Zhang S Zhang Y Liang Y. PD-L1 on dendritic cells attenuates T cell activation and regulates response to immune checkpoint blockade. Nat. Commun. (2020) 11:1–8. 10.1038/s41467-020-18570-x 32973173 PMC7518441

[125] Laoui D Keirsse J Morias Y Van Overmeire E Geeraerts X Elkrim Y. The tumour microenvironment harbours ontogenically distinct dendritic cell populations with opposing effects on tumour immunity. Nat. Commun. (2016) 7:1–17. 10.1038/ncomms13720 28008905 PMC5196231

[126] Binnewies M Mujal AM Pollack JL Combes AJ Hardison EA Barry KC. Unleashing type-2 dendritic cells to drive protective antitumor CD4+ T cell immunity. Cell. (2019) 177:556–71.e516. 10.1016/j.cell.2019.02.005 30955881 PMC6954108

[127] Norian LA Rodriguez PC O'mara LA Zabaleta J Ochoa AC Cella M. Tumor-infiltrating regulatory dendritic cells inhibit CD8+ T cell function via L-arginine metabolism. Cancer Res. (2009) 69:3086–94. 10.1158/0008-5472.CAN-08-2826 19293186 PMC2848068

[128] Segura E Touzot M Bohineust A Cappuccio A Chiocchia G Hosmalin A. Human inflammatory dendritic cells induce Th17 cell differentiation. Immunity. (2013) 38:336–48. 10.1016/j.immuni.2012.10.018 23352235

[129] Shinde P Fernandes S Melinkeri S Kale V Limaye L. Compromised functionality of monocyte-derived dendritic cells in multiple myeloma patients may limit their use in cancer immunotherapy. Sci. Rep. (2018) 8:1–11. 10.1038/s41598-018-23943-w 29632307 PMC5890285

[130] Oosterhoff D Lougheed S Van De Ven R Lindenberg J Van Cruijsen H Hiddingh L. Tumor-mediated inhibition of human dendritic cell differentiation and function is consistently counteracted by combined p38 MAPK and STAT3 inhibition. Oncoimmunology. (2012) 1:649–58. 10.4161/onci.20365 22934257 PMC3429569

[131] Goldszmid RS Caspar P Rivollier A White S Dzutsev A Hieny S. NK cell-derived interferon-γ orchestrates cellular dynamics and the differentiation of monocytes into dendritic cells at the site of infection. Immunity. (2012) 36:1047–59. 10.1016/j.immuni.2012.03.026 22749354 PMC3412151

[132] Shin K-S Jeon I Kim B-S Kim I-K Park Y-J Koh C-H. Monocyte-derived dendritic cells dictate the memory differentiation of CD8+ T cells during acute infection. Front. Immunol. (2019) 10:1887. 10.3389/fimmu.2019.01887 31474983 PMC6706816

[133] Wang Y Xiang Y Xin VW Wang X-W Peng X-C Liu X-Q. Dendritic cell biology and its role in tumor immunotherapy. J. Hematol. Oncol. (2020) 13:1–18. 10.1186/s13045-020-00939-6 32746880 PMC7397618

[134] Ward S Casey D Labarthe M-C Whelan M Dalgleish A Pandha H. Immunotherapeutic potential of whole tumour cells. Cancer Immunol. Immunother. (2002) 51:351–7. 10.1007/s00262-002-0286-2 12192534 PMC11033012

[135] Turnis ME Rooney CM. Enhancement of dendritic cells as vaccines for cancer. Immunotherapy. (2010) 2:847–62. 10.2217/imt.10.56 21091116 PMC3433954

[136] Draube A Klein-Gonzalez N Mattheus S Brillant C Hellmich M Engert A. Dendritic cell based tumor vaccination in prostate and renal cell cancer: a systematic review and meta-analysis. PloS ONE. (2011) 6:18801. 10.1371/journal.pone.0018801 21533099 PMC3080391

[137] Tirosh I Venteicher AS Hebert C Escalante LE Patel AP Yizhak K. Single-cell RNA-seq supports a developmental hierarchy in human oligodendroglioma. Nature. (2016) 539:309–13. 10.1038/nature20123 27806376 PMC5465819

[138] Fan J Slowikowski K Zhang F. Single-cell transcriptomics in cancer: computational challenges and opportunities. Exp. Mol. Med. (2020) 52:1452–65. 10.1038/s12276-020-0422-0 32929226 PMC8080633

[139] Ramsköld D Luo S Wang Y-C Li R Deng Q Faridani OR . Full-length mRNA-Seq from single-cell levels of RNA and individual circulating tumor cells. Nat. Biotechnol. (2012) 30:777–82. 10.1038/nbt.2282 22820318 PMC3467340

[140] Gilboa E Vieweg J. Cancer immunotherapy with mRNA-transfected dendritic cells. Immunol. Rev. (2004) 199:251–63. 10.1111/j.0105-2896.2004.00139.x 15233739

[141] Van Nuffel AM Benteyn D Wilgenhof S Pierret L Corthals J Heirman C. Dendritic cells loaded with mRNA encoding full-length tumor antigens prime CD4+ and CD8+ T cells in melanoma patients. Mol. Ther. (2012) 20:1063–74. 10.1038/mt.2012.11 22371843 PMC3345975

[142] Su Z Dannull J Yang BK Dahm P Coleman D Yancey D. Telomerase mRNA-transfected dendritic cells stimulate antigen-specific CD8+ and CD4+ T cell responses in patients with metastatic prostate cancer. J. Immunol. (2005) 174:3798–807. 10.4049/jimmunol.174.6.3798 15749921

[143] Su Z Vieweg J Weizer AZ Dahm P Yancey D Turaga V. Enhanced induction of telomerase-specific CD4+ T cells using dendritic cells transfected with RNA encoding a chimeric gene product. Cancer Res. (2002) 62:5041–8. 12208759

[144] Cheever MA Higano CS. PROVENGE (Sipuleucel-T) in prostate cancer: the first FDA-approved therapeutic cancer vaccine. Clin. Cancer Res. (2011) 17:3520–6. 10.1158/1078-0432.CCR-10-3126 21471425

[145] Anguille S Smits EL Lion E Van Tendeloo VF Berneman ZN. Clinical use of dendritic cells for cancer therapy. Lancet Oncol. (2014) 15:e257–67. 10.1016/S1470-2045(13)70585-0 24872109

[146] Alvarez-Dominguez C Calderón-Gonzalez R Terán-Navarro H Salcines-Cuevas D Garcia-Castaño A Freire J. Dendritic cell therapy in melanoma. Ann. Transl. Med. (2017) 5:386. 10.21037/atm.2017.06.13 29114544 PMC5653516

[147] Oh SA Wu D-C Cheung J Navarro A Xiong H Cubas R. PD-L1 expression by dendritic cells is a key regulator of T-cell immunity in cancer. Nat. Cancer. (2020) 1:681–91. 10.1038/s43018-020-0075-x 35122038

[148] Van Der Waart AB Fredrix H Van Der Voort R Schaap N Hobo W Dolstra H. siRNA silencing of PD-1 ligands on dendritic cell vaccines boosts the expansion of minor histocompatibility antigen-specific CD8+ T cells in NOD/SCID/IL2Rg (null) mice. Cancer Immunol. Immunother. (2015) 64:645–54. 10.1007/s00262-015-1668-6 25724840 PMC4412509

[149] Hobo W Novobrantseva TI Fredrix H Wong J Milstein S Epstein-Barash H. Improving dendritic cell vaccine immunogenicity by silencing PD-1 ligands using siRNA-lipid nanoparticles combined with antigen mRNA electroporation. Cancer Immunol. Immunother. (2013) 62:285–97. 10.1007/s00262-012-1334-1 22903385 PMC11028421

[150] Flatekval GF Sioud M. Modulation of dendritic cell maturation and function with mono-and bifunctional small interfering RNAs targeting indoleamine 2, 3-dioxygenase. Immunology. (2009) 128:e837–48. 10.1111/j.1365-2567.2009.03093.x 19740345 PMC2753901

[151] Zheng X Koropatnick J Chen D Velenosi T Ling H Zhang X. Silencing IDO in dendritic cells: a novel approach to enhance cancer immunotherapy in a murine breast cancer model. Int. J. Cancer. (2013) 132:967–77. 10.1002/ijc.27710 22870862

[152] Sioud M Sæbøe-Larssen S Hetland TE Kærn J Mobergslien A Kvalheim G. Silencing of indoleamine 2, 3-dioxygenase enhances dendritic cell immunogenicity and antitumour immunity in cancer patients. Int. J. Oncol. (2013) 43:280–8. 10.3892/ijo.2013.1922 23620105

[153] Chen D Koropatnick J Jiang N Zheng X Zhang X Wang H. Targeted siRNA silencing of indoleamine 2, 3-dioxygenase in antigen-presenting cells using mannose-conjugated liposomes: a novel strategy for treatment of melanoma. J. Immunotherapy. (2014) 37:123–34. 10.1097/CJI.0000000000000022 24509175

[154] Endo R Nakamura T Kawakami K Sato Y Harashima H. The silencing of indoleamine 2, 3-dioxygenase 1 (IDO1) in dendritic cells by siRNA-loaded lipid nanoparticles enhances cell-based cancer immunotherapy. Sci. Rep. (2019) 9:1–11. 10.1038/s41598-019-47799-w 31383907 PMC6683295

[155] Flaherty KT Puzanov I Kim KB Ribas A Mcarthur GA Sosman JA. Inhibition of mutated, activated BRAF in metastatic melanoma. N. Engl. J. Med. (2010) 363:809–19. 10.1056/NEJMoa1002011 20818844 PMC3724529

[156] Sosman JA Kim KB Schuchter L Gonzalez R Pavlick AC Weber JS. Survival in BRAF V600–mutant advanced melanoma treated with vemurafenib. N. Engl. J. Med. (2012) 366:707–14. 10.1056/NEJMoa1112302 22356324 PMC3724515

[157] Chapman PB Hauschild A Robert C Haanen JB Ascierto P Larkin J. Improved survival with vemurafenib in melanoma with BRAF V600E mutation. N. Engl. J. Med. (2011) 364:2507–16. 10.1056/NEJMoa1103782 21639808 PMC3549296

[158] Johannessen CM Boehm JS Kim SY Thomas SR Wardwell L Johnson LA. COT drives resistance to RAF inhibition through MAP kinase pathway reactivation. Nature. (2010) 468:968–72. 10.1038/nature09627 21107320 PMC3058384

[159] Bardia A Gounder M Rodon J Janku F Lolkema MP Stephenson JJ. Phase Ib study of combination therapy with MEK inhibitor binimetinib and phosphatidylinositol 3-kinase inhibitor buparlisib in patients with advanced solid tumors with RAS/RAF alterations. Oncologist. (2020) 25:e160. 10.1634/theoncologist.2019-0297 31395751 PMC6964137

[160] Ascierto PA Schadendorf D Berking C Agarwala SS Van Herpen CM Queirolo P. MEK162 for patients with advanced melanoma harbouring NRAS or Val600 BRAF mutations: a non-randomised, open-label phase 2 study. Lancet Oncol. (2013) 14:249–56. 10.1016/S1470-2045(13)70024-X 23414587

[161] Margolin K Longmate J Baratta T Synold T Christensen S Weber J. CCI-779 in metastatic melanoma: a phase II trial of the California Cancer Consortium. Cancer. (2005) 104:1045–8. 10.1002/cncr.21265 16007689

[162] Ernst DS Eisenhauer E Wainman N Davis M Lohmann R Baetz T. Phase II study of perifosine in previously untreated patients with metastatic melanoma. Invest. New Drugs. (2005) 23:569–75. 10.1007/s10637-005-1157-4 16034524

[163] Tinder TL Subramani DB Basu GD Bradley JM Schettini J Million A. MUC1 enhances tumor progression and contributes toward immunosuppression in a mouse model of spontaneous pancreatic adenocarcinoma. J. Immunol. (2008) 181:3116–25. 10.4049/jimmunol.181.5.3116 18713982 PMC2625292

[164] Gross S Erdmann M Haendle I Voland S Berger T Schultz E. Twelve-year survival and immune correlates in dendritic cell–vaccinated melanoma patients. JCI Insight. (2017) 2:e91438. 10.1172/jci.insight.91438 28422751 PMC5396520

[165] Loveland BE Zhao A White S Gan H Hamilton K Xing P-X. Mannan-MUC1–pulsed dendritic cell immunotherapy: a phase I trial in patients with adenocarcinoma. Clin. Cancer Res. (2006) 12:869–77. 10.1158/1078-0432.CCR-05-1574 16467101

[166] Lepisto AJ Moser AJ Zeh H Lee K Bartlett D Mckolanis JR. A phase I/II study of a MUC1 peptide pulsed autologous dendritic cell vaccine as adjuvant therapy in patients with resected pancreatic and biliary tumors. Cancer Ther. (2008) 6:955–64. 19129927 PMC2614325

[167] Thurner B Haendle I Röder C Dieckmann D Keikavoussi P Jonuleit H. Vaccination with mage-3A1 peptide–pulsed mature, monocyte-derived dendritic cells expands specific cytotoxic T cells and induces regression of some metastases in advanced stage IV melanoma. J. Exp. Med. (1999) 190:1669–78. 10.1084/jem.190.11.1669 10587357 PMC2195739

[168] Krause SW Neumann C Soruri A Mayer S Peters JH Andreesen R. The treatment of patients with disseminated malignant melanoma by vaccination with autologous cell hybrids of tumor cells and dendritic cells. J. Immunother. (2002) 25:421–8. 10.1097/00002371-200209000-00006 12218780

[169] Redman BG Chang AE Whitfield J Esper P Jiang G Braun T. Phase Ib trial assessing autologous, tumor-pulsed dendritic cells as a vaccine administered with or without IL-2 in patients with metastatic melanoma. J. Immunother. (2008) 31:591. 10.1097/CJI.0b013e31817fd90b 18528294 PMC2642589

[170] Lesterhuis WJ Schreibelt G Scharenborg NM Brouwer HM-LH Gerritsen M-JP Croockewit S. Wild-type and modified gp100 peptide-pulsed dendritic cell vaccination of advanced melanoma patients can lead to long-term clinical responses independent of the peptide used. Cancer Immunol. Immunother. (2011) 60:249–60. 10.1007/s00262-010-0942-x 21069321 PMC11029288

[171] Lotfinejad P Asghari Jafarabadi M Abdoli Shadbad M Kazemi T Pashazadeh F Sandoghchian Shotorbani S. Prognostic role and clinical significance of tumor-infiltrating lymphocyte (TIL) and programmed death ligand 1 (PD-L1) expression in triple-negative breast cancer (TNBC): a systematic review and meta-analysis study. Diagnostics. (2020) 10:704. 10.3390/diagnostics10090704 32957579 PMC7554852

[172] Aierken N Shi H-J Zhou Y Shao N Zhang J Shi Y. High PD-L1 expression is closely associated with tumor-infiltrating lymphocytes and leads to good clinical outcomes in Chinese triple negative breast cancer patients. Int. J. Biol. Sci. (2017) 13:1172. 10.7150/ijbs.20868 29104508 PMC5666332

[173] Mori H Kubo M Yamaguchi R Nishimura R Osako T Arima N. The combination of PD-L1 expression and decreased tumor-infiltrating lymphocytes is associated with a poor prognosis in triple-negative breast cancer. Oncotarget. (2017) 8:15584. 10.18632/oncotarget.14698 28107186 PMC5362507

[174] Bulgarelli J Tazzari M Granato AM Ridolfi L Maiocchi S De Rosa F. Dendritic cell vaccination in metastatic melanoma turns “non-T cell inflamed” into “T-cell inflamed” tumors. Front. Immunol. (2019) 10:2353. 10.3389/fimmu.2019.02353 31649669 PMC6794451

[175] Larkin J Chiarion-Sileni V Gonzalez R Grob JJ Cowey CL Lao CD. Combined nivolumab and ipilimumab or monotherapy in untreated melanoma. N. Engl. J. Med. (2015) 373:23–34. 10.1056/NEJMoa1504030 26027431 PMC5698905

[176] Schachter J Ribas A Long GV Arance A Grob J-J Mortier L. Pembrolizumab versus ipilimumab for advanced melanoma: final overall survival results of a multicentre, randomised, open-label phase 3 study (KEYNOTE-006). Lancet. (2017) 390:1853–62. 10.1016/S0140-6736(17)31601-X 28822576

[177] Li J Gu J. Efficacy and safety of PD-1 inhibitors for treating advanced melanoma: a systematic review and meta-analysis. Immunotherapy. (2018) 10:1293–302. 10.2217/imt-2018-0116 30474476

[178] Hao C Tian J Liu H Li F Niu H Zhu B. Efficacy and safety of anti-PD-1 and anti-PD-1 combined with anti-CTLA-4 immunotherapy to advanced melanoma: a systematic review and meta-analysis of randomized controlled trials. Medicine. (2017) 96:e7325. 10.1097/MD.0000000000007325 28658143 PMC5500065

[179] Yang H Shen K Zhu C Li Q Zhao Y Ma X. Safety and efficacy of durvalumab (MEDI4736) in various solid tumors. Drug Design Dev. Ther. (2018) 12:2085. 10.2147/DDDT.S162214 30013326 PMC6038862

[180] Planchard D Yokoi T Mccleod MJ Fischer JR Kim Y-C Ballas M. A phase III study of durvalumab (MEDI4736) with or without tremelimumab for previously treated patients with advanced NSCLC: rationale and protocol design of the ARCTIC study. Clin. Lung Cancer. (2016) 17:232–6.e231. 10.1016/j.cllc.2016.03.003 27265743

[181] Verschraegen CF Jerusalem G Mcclay EF Iannotti N Redfern CH Bennouna J. Efficacy and safety of first-line avelumab in patients with advanced non-small cell lung cancer: results from a phase Ib cohort of the JAVELIN solid tumor study. J. Immunother. Cancer. (2020) 8:e001064. 10.1136/jitc-2020-001064 32907924 PMC7481079

[182] Keilholz U Mehnert JM Bauer S Bourgeois H Patel MR Gravenor D. Avelumab in patients with previously treated metastatic melanoma: phase 1b results from the JAVELIN Solid Tumor trial. J. Immunother. Cancer. (2019) 7:12. 10.1186/s40425-018-0459-y 30651126 PMC6335739

[183] Robert C Schachter J Long GV Arance A Grob JJ Mortier L. Pembrolizumab versus ipilimumab in advanced melanoma. N. Engl. J. Med. (2015) 372:2521–32. 10.1056/NEJMoa1503093 25891173

[184] Kirchberger MC Moreira A Erdmann M Schuler G Heinzerling L. Real world experience in low-dose ipilimumab in combination with PD-1 blockade in advanced melanoma patients. Oncotarget. (2018) 9:28903. 10.18632/oncotarget.25627 29988983 PMC6034742

[185] Jochems C Fantini M Fernando RI Kwilas AR Donahue RN Lepone LM. The IDO1 selective inhibitor epacadostat enhances dendritic cell immunogenicity and lytic ability of tumor antigen-specific T cells. /*Oncotarget*. (2016) 7:37762. 10.18632/oncotarget.9326 27192116 PMC5122347

[186] Daud A Saleh MN Hu J Bleeker JS Riese MJ Meier R. Epacadostat plus nivolumab for advanced melanoma: updated phase 2 results of the ECHO-204 study. Am. Soc. Clin. Oncol. (2018) 36:9511. 10.1200/JCO.2018.36.15_suppl.9511

[187] Zakharia Y Rixe O Ward JH Drabick JJ Shaheen MF Milhem MM. Phase 2 trial of the IDO pathway inhibitor indoximod plus checkpoint inhibition for the treatment of patients with advanced melanoma. Am. Soc. Clin. Oncol. (2018) 36:9512. 10.1200/JCO.2018.36.15_suppl.9512

[188] Gibney GT Hamid O Lutzky J Olszanski AJ Mitchell TC Gajewski TF. Phase 1/2 study of epacadostat in combination with ipilimumab in patients with unresectable or metastatic melanoma. J. Immunother. Cancer. (2019) 7:80. 10.1186/s40425-019-0562-8 30894212 PMC6425606

[189] Long GV Dummer R Hamid O Gajewski TF Caglevic C Dalle S. Epacadostat plus pembrolizumab versus placebo plus pembrolizumab in patients with unresectable or metastatic melanoma (ECHO-301/KEYNOTE-252): a phase 3, randomised, double-blind study. Lancet Oncol. (2019) 20:1083–97. 10.1016/S1470-2045(19)30274-8 31221619

